# Mitral Valve Repair: Optimal Annual Case Volume for Surgery

**DOI:** 10.1016/j.jacadv.2025.101589

**Published:** 2025-02-10

**Authors:** Irbaz Hameed, Adham Ahmed, Christina Waldron, Percy T. Algarate, Michal Kawczynski, Maurish Fatima, Amnah Alhazmi, Samantha Colon, Alexandria Brackett, Samuel Heuts, Peyman Sardari Nia, Mario Gaudino, Vinay Badhwar, Arnar Geirsson

**Affiliations:** a Division of Cardiac Surgery, Department of Surgery, Yale School of Medicine, New Haven, Connecticut, USA; b Department of Cardiothoracic Surgery, Maastricht University Medical Center, Maastricht, the Netherlands; c Cardiovascular Research Institute Maastricht (CARIM), Maastricht University, Maastricht, the Netherlands; d Department of Cardiothoracic Surgery, New York Presbyterian-Weill Cornell Medicine, New York, New York, USA; e Department of Cardiovascular and Thoracic Surgery, West Virginia University, Morgantown, West Virginia, USA; f Division of Cardiac Surgery, New York Presbyterian-Columbia University School of Medicine, New York, New York, USA

**Keywords:** annuloplasty, mitral valve, repair, volume

## Abstract

**Background:**

Despite strong recommendations from multiple societies to pursue durable mitral valve repair (MVr), repair rates and outcomes remain inconsistent across the world. This is partly due to limited surgeon and center experience and lack of centralization of care for this technically challenging operation.

**Objectives:**

The authors evaluate the association between annual case volume and contemporary long-term outcomes of patients undergoing isolated MVr.

**Methods:**

A systematic literature search was performed to identify contemporary studies on isolated MVr in adults from January 2013 to November 2023. The primary outcomes were long-term survival, freedom from reoperation, and freedom from recurrent mitral regurgitation (moderate-severe). A novel meta-analytic volume-outcome approach using reconstructed Kaplan-Meier-derived individual patient data from the original studies was used. A frailty Cox model was applied to study volume-outcome relationships. Studies were pooled for each reported outcome and divided into 3 tertiles (T1-3) based on the annual case volume and number of patients of each center.

**Results:**

A total of 14,070 patients from 60 studies were pooled. Sixteen studies (6,099 patients) reported long-term survival. The overall pooled 10-year survival was 70.8% (95% CI: 68.9%-72.8%). Compared to lower volume centers, centers performing >38 cases/y were associated with significantly improved long-term survival (HR: 0.42; 95% CI: 0.36-0.49; *P* < 0.001). For degenerative mitral valve disease, a volume cutoff of >45 cases/y was associated with significantly improved long-term survival (HR: 0.40; 95% CI: 0.32-0.49; *P* < 0.001). Twelve studies (4,230 patients) reported long-term freedom from reoperation and 10 studies (2,470 patients) reported Kaplan-Meier-derived long-term freedom from recurrent mitral regurgitation data, respectively. The overall pooled 10-year freedom from reoperation was 90.2% (95% CI: 88.1%-92.4%), while the overall pooled 10-year freedom from recurrent mitral regurgitation was 72.7% (95% CI: 68.9%-76.8%). Centers performing >45 cases/y (HR: 0.61; 95% CI: 0.44-0.84; *P* = 0.003) and >70 cases/y (HR: 0.64; 95% CI: 0.42-0.98; *P* = 0.042) were associated with significantly improved long-term freedom from recurrent mitral regurgitation and freedom from reoperation, respectively. For degenerative mitral disease, >45 cases/y was associated with significantly improved freedom from recurrent mitral regurgitation (HR: 0.51; 95% CI: 0.36-0.72; *P* < 0.001); the volume outcome association for freedom from reoperation was not statistically significant (*P* = 0.58).

**Conclusions:**

Our study validates volume cutoffs associated with optimal long-term outcomes following isolated MVr. We found MVr volumes of >38 cases/y, 45 cases/y, and >70 cases/y to be associated with significantly improved long-term survival, durability of repair, and freedom from reoperation, respectively. These findings may define experienced centers and surgeons for patients requiring MVr for primary/degenerative mitral valve disease.

Mitral valve repair (MVr) is the preferred treatment for severe mitral regurgitation in patients with primary/degenerative mitral disease.^1^ Despite strong recommendations from multiple societies to pursue durable repair, especially in asymptomatic patients, repair rates and outcomes remain inconsistent across the world.^2^ The rate of mitral valve replacement in patients with primary/degenerative mitral valve disease also remains unacceptably high.^3^^,^^4^ This has been partly attributed to limited surgeon and institutional experience.^5^^,^^6^ Consequently, various volume cutoffs have been proposed to describe experienced mitral repair centers and surgeons to centralize care.^7^ These range from 10 cases/y to 50 cases/y.^8^^,^^9^ These benchmarks are based on registry/multicenter data with limitations such as short follow-up, inclusion of patients with heterogeneous mitral pathology, nondistinction between MVr and replacement, and other limitations of administrative data sets.^5^^,^^6^

**Key Question:** What is the optimal annual case volume for isolated MVr?

**Key Finding:** Compared to lower volume centers, those performing >38 cases/y, >70 cases/y, and >45 cases/y were associated with significantly lower rates of long-term mortality, reoperation, and greater than mild mitral regurgitation recurrence.

**Take-Home Message:** Our study validates volume cutoffs associated with optimal long-term outcomes following isolated MVr. These findings may define experienced centers and surgeons for patients requiring MVr for primary/degenerative mitral valve disease.

By using a novel meta-analytic method to define volume-outcome relationship,^10^ we evaluated the association between annual case volume and contemporary long-term outcomes of patients undergoing isolated MVr to validate these volume cutoffs.

## Materials and methods

### Search strategy

The study protocol was prospectively registered in the PROSPERO (International Prospective Register of Systematic Reviews) registry for systematic reviews (CRD42023473897). Informed patient consent and ethical board approval were not required as individual patient data were not used. A medical librarian (A.B.) performed a comprehensive literature search on medical databases to identify contemporary observational studies and randomized control trials reporting outcomes following isolated MVr outcomes in patients >18 years of age. The search was performed on November 14, 2023, on the Ovid MEDLINE, Ovid EMBASE (1974 to present), and the Cochrane Library (Wiley) databases and restricted to studies published from January 1, 2013.^11^ The full search strategy is reported in Supplemental Table 1.

### Study selection

Following deduplication, 2 authors (A.A. and C.W.) independently reviewed the titles and abstracts of each citation for eligibility (Supplemental Figure 1).^12^ Any disagreements were resolved by a third more senior investigator (I.H.). Articles were eligible for inclusion if they were a randomized or nonrandomized study reporting at least one of the prespecified primary outcomes (long-term survival, freedom from reoperation, or freedom from recurrent mitral regurgitation) following isolated MVr. Studies including patients undergoing redo MVr, mitral valve replacement, concomitant valve surgery, and/or coronary artery bypass surgery were excluded. We favored single-center studies for accurate estimation of centers' annual MVr case volume. Case series, laboratory studies, non-English studies, editorials, and review papers were excluded. We additionally manually searched the references of eligible studies for other relevant citations. Secondary screening was completed by 2 authors independently (C.W. and P.T.A.), and discrepancies were resolved by discussion with a third investigator (I.H.). In cases of overlapping patient cohorts or duplicate studies from the same center, we included only the largest series.

### Data extraction

Two investigators (A.A. and C.W.) independently performed data extraction, with data reviewed by a third investigator (I.H.) for accuracy. The following data were extracted: study/center information (year of publication, period of inclusion, sample size, name of institution, and country) (Table 1), patient baseline demographics, and comorbidities (EuroSCORE, Society of Thoracic Surgeons risk score, NYHA functional class, hypertension, dyslipidemia, diabetes mellitus, smoking status, prior stroke, prior myocardial infarction, prior cardiac operations, peripheral vascular disease, chronic obstructive pulmonary disease, aortic regurgitation, renal failure, heart failure), and presenting etiology (Supplemental Table 2). Adapting previous methodology,^10^ annual case volume was calculated by dividing the total number of cases by the years of patient inclusion duration of each study.

**Table 1:** Summary of Included Studies

First Author/Year	Center	Country	Period of Inclusion	Annual Case Volume	Follow-Up Duration	Sample Size
Afshar/2023	Imam Ali Hospital	Iran	2014-2021	9.88	-	75
Agnino/2022	Humanitas Gavazzeni Hospital	Italy	2019-2021	26.17	-	59
Agnino/2019	Humanitas Gavazzeni Hospital	Italy	2012-2017	7.05	-	37
Ascaso/2023	Hospital Clínico de Barcelona	Spain	2011-2020	33.30	6 y	300
Atluri/2016	University of Pennsylvania	USA	2012-2013	105.71	-	159
Balachandran/2020	Mayo Clinic	USA	2008-2015	105.37	-	720
Bellitti/2014	Monaldi Hospital	Italy	2000-2011	12.72	12 y	140
Berdajs/2023	University of Basel	Switzerland	2009-2021	29.59	10 y	365
Chemtob/2022	Cleveland Clinic	USA	2014-2019	199.60	-	998
Chi/2014	National Taiwan University Hospital	Taiwan	2012-2013	6.26	-	12
D'Onofrio/2022	Padova University Hospital	Italy	2010-2018	19.73	5 y	176
El-Andari/2021	University of Alberta Hospital	Canada	2004-2018	50.03	-	701
Franke/2022	Robert Bosch Hospital	Germany	2019-2021	39.64	-	96
Garatti/2018	IRCCS Policlinico San Donato	Italy	2002-2008	19.36	-	134
Fuster/2014	University General Hospital of Valencia	Spain	2009-2012	19.44	-	68
Gerber/2019	American Heart of Poland	Poland	2012-2017	22.78	-	114
Goldstone/2015	University of Pennsylvania Health System	USA	2002-2011	58.30	12.5 y	525
Götte/2022	Heart and Diabetes Center NRW	Germany	2009-2018	27.27	10 y	257
Grapsa/2015	Hammersmith Hospital	UK	2008-2012	15.99	-	64
Grinberg/2019	Hôpital Louis Pradel, Lyon Medical School	France	2016-2017	4.20	-	7
Güllü/2021	Acibadem Mehmet Ali Aydınlar University School of Medicine	Turkey	2010-2019	6.31	-	60
Hashim/2022	Hartford Hospital	USA	2016-2021	60.41	5 y	287
Hayashi/2021	Chiba West General Hospital	Japan	2014-2019	19.69	-	100
Hu/2021	Wuhan Union Hospital	China	2003-2018	41.50	12 y	623
Huang/2013	Guangdong General Hospital	China	2008-2010	24.00	-	48
Javadikasgari/2017	Cleveland Clinic Hospital System	USA	2006-2013	79.48	-	623
Kamiya/2017	Heinrich Heine University	Germany	2009-2013	5.50	-	22
Kitai/2014	Kobe City Medical Center General Hospital	Japan	1996-2010	33.76	-	473
Kurlansky/2023	Columbia University Irving Medical Center	USA	1985-2005	20.74	30 y	434
Lange/2017	Munich School of Medicine	Germany	2000-2010	69.26	10 y	745
Lee/2018	Linkou Chang-Gung Memorial Hospital	Taiwan	2005-2015	3.80	-	38
Levy/2022	Cardiovascular and Thoracic Centre of Monaco	Monaco	NR (36-mo)	13.00	-	39
Li/2022	Beijing Anzhen Hospital	China	2016-2021	21.81	-	120
Lio/2017	Istituto Clinico Sant'Ambrogio	Italy	2014-2016	13.04	-	25
Ma/2019	Shanghai Chest Hospital	China	2006-2016	38.98	10.4 y	390
Magruder/2016	The Johns Hopkins University	USA	1997-2014	13.11	10 y	223
Laham/2023	University Hospital of Essen	Germany	2010-2016	9.84	8 y	64
Mihaljevic/2013	Cleveland Clinic Abu Dhabi	Dubai	2007-2010	129.28	-	334
Moscarelli/2018	Heart Hospital “Gaetano Pasquinucci”	Italy	2015-2016	35.30	6 y	62
Moscarelli/2021	Maria Cecilia Hospital, Anthea Hospital	Italy	2012-2018	9.00	6 y	54
Muneretto/2015	University of Brescia Medical School	Italy	2009-2012	15.80	-	50
Murzi/2014	Heart Hospital “Gaetano Pasquinucci”	Italy	2007-2013	99.08	10.4 y	595
Noack/2019	Heart Center Leipzig	Germany	1997-2015	30.93	10 y	557
Panos/2015	Geneva University Hospitals	Switzerland	2008-2013	85.11	7 y	426
Paranskaya/2013	University Hospital of Rostock	Germany	2010-2011	15.58	-	26
Perin/2019	Liverpool Heart and Chest Hospital	UK	3/11-3/16	21.58	-	108
Petolat/2023	Marseille University Hospital Timone	France	2014-2019	39.58	-	198
Ramzy/2014	Cedars-Sinai Medical Center	USA	2005-2012	40.87	-	300
Salihi/2019	Sakarya University Training and Research Hospital	Turkey	2015-2017	12.98	4.2 y	26
Shibata/2015	Osaka City General Hospital	Japan	2008-2013	33.73	6 y	180
Smith/2016	University of North Dakota School of Medicine and Health Sciences	USA	2006-2010	15.75	-	63
Solari/2019	Saint Luc University Clinic	Belgium	1991-2015	6.37	15 y	155
Tomšič/2019	Leiden University Medical Center	Netherlands	2002-2015	45.03	8 y	627
Uchimuro/2014	Sakakibara Heart Institute	Japan	2004-2008	29.13	-	119
Vairo/2023	Azienda Ospedaliero-Universitaria Città della Salute e della Scienza di Torino	Italy	2015-2021	12.14	-	72
van Leeuwen/2013	Erasmus University Medical Centre	Netherlands	1991-2005	3.15	-	46
Van Praet/2022	German Heart Institute Berlin	Germany	2014-2020	85.34	-	491
Wang/2016	Chinese People's Liberation Army General Hospital	China	2007-2015	10.59	-	84
Westhofen/2016	University Heart Center Hamburg	Germany	2011-2015	22.62	-	98
Zorinas/2019	Vilnius University Hospital	Lithuania	2011-2018	11.13	-	78

Operative details, including repair strategy, operation duration, cardiopulmonary bypass/cross-clamp duration, and the use minimally invasive/robotic approach were also recorded and tabulated (Supplemental Table 3).

### Postoperative outcomes

The primary outcomes for our analysis were long-term survival, freedom from reoperation, and freedom from recurrent mitral regurgitation (moderate-severe). The secondary outcomes were early postoperative/in-hospital/30-day mortality, stroke, major bleeding, and renal failure. The definitions of the included outcomes matched those reported in the original studies (Supplemental Table 6). Subgroup analyses were performed for each of our outcomes utilizing studies that reported on patients with degenerative mitral valve disease, defined as mitral regurgitation secondary to primary valvular pathology, including but not limited to myxomatous degeneration of the mitral valve leaflets, chordae tendineae elongation or rupture, and/or papillary muscle pathology.

### Study quality/quality of evidence assessment

We assessed the quality of observational cohort studies using the Newcastle–Ottawa Scale for observational studies.^13^ Trial quality was assessed using the Cochrane Collaboration's tool for assessing risk of bias in randomized trials^14^ (Supplemental Tables 4 and 5).

### Data synthesis

All statistical analyses were performed in R, version 3.6.0 (R foundation)^15^ using the “metafor,” “meta,” “rms,” “mgcv,” “survival,” “survminer,” “maps,” “ggplot2,” “path-viewr,” and “discfrail” packages (Supplemental Table 1). Studies were pooled and divided into 3 tertiles (T1-3) based on the annual case volume and number of patients of each center, which differed for the outcomes. Baseline and peri-operative variables were compared between the tertiles using the chi-square test and 1-way analysis of variance test for categorical and continuous outcomes, respectively. Postoperative outcomes were pooled using the inverse variance method and random effects model. Heterogeneity was assessed using The Higgins' and Thompson's I^2^ statistic,^16^ and classified as low (I^2^ = 0%-25%), moderate (I^2^ = 25%-50%), or high (I^2^ >50%) and publication bias was assessed using the Egger's test along with visual evaluation in funnel plots.

For the primary outcomes, a meta-analysis of reconstructed Kaplan-Meier-derived individual patient data was performed using Kaplan-Meier curves from the original studies.^17^ The reconstructed curves were presented based on prespecified volume tertiles. To assess the volume/outcome relationship, a frailty Cox model was applied with adjustment for center, age, sex, median inclusion year, and geographic continent in line with prior methodology.^10^ Adjustments for clinical and study-associated heterogeneity were performed by including a random effects term (study variable) in the Cox frailty model. The proportional hazard assumption was visually assessed and tested using Schoenfeld residuals. In cases where proportionality of hazard was violated, a restricted mean survival time (RMST) analysis was performed to assess the difference between volume tertiles.

For secondary outcomes, potential nonlinear relations between annual case volume (taken as a continuous variable) and short-term outcomes were assessed using a restricted cubic spline model using 3 knots and presented graphically via nonlinear mixed effects model. Center weights were assigned inversely based on data variance. In case of statistically significant nonlinear association, the optimal annual case volume cutoff for short-term outcomes was specified using an “elbow of the curve” method, which allowed us to determine the plateau point of the curve,^18^^,^^19^ at which an increase in case volume would not lead to a further reduction in the adverse event.

## Results

### Study characteristics

A total of 2,620 full texts were screened, following which data from 60 eligible studies were evaluated for analysis. (Supplemental Figure 1, Supplemental Table 7). Eleven studies were from U.S. centers, 13 from Italy, 4 from Japan, and the rest from other countries. The details of the included studies are reported in Table 1.

A total of 14,070 adult patients from 58 unique centers undergoing isolated MVr were evaluated. Study sample sizes ranged from 7 to 1,006 patients and annual case volumes ranged from 3.15 to 199.60 cases/y. The mean age of patients ranged from 42.3 to 80.0 years old, with female patients constituting 10.0% to 61.7% of study cohorts. Study quality assessment is reported in Supplemental Tables 4 and 5.

### Primary outcomes

#### Long-term survival

Sixteen studies (n = 6,099) reported long-term survival data. The overall pooled 10-year survival was 70.8% (95% CI: 68.9%-72.8%). Centers were divided into the following individual volume tertiles for assessment: T1 = 0 to 38 cases/y, T2 = 38 to 69 cases/y, and T3 = 69 to 100 cases/y. There was a significant association between center volume and long-term survival (T2 vs T1: HR: = 0.42; 95% CI: 0.36-0.49; *P* < 0.001, and T3 vs T1: HR: 0.40; 95% CI: 0.34-0.48; *P* < 0.001) (test for proportionality of hazard; *P* = 0.87) (Central Illustration). Furthermore, in a subgroup analysis of 8 studies (n = 3,015) undergoing isolated MVr for degenerative mitral disease, centers performing >45 cases/y were associated with significantly longer survival compared to centers performing <30 cases/y (HR: 0.40; 95% CI: 0.32-0.49; *P* < 0.001) (test for proportionality of hazard; *P* = 0.08) (Supplemental Figure 2).

**Central Illustration:**
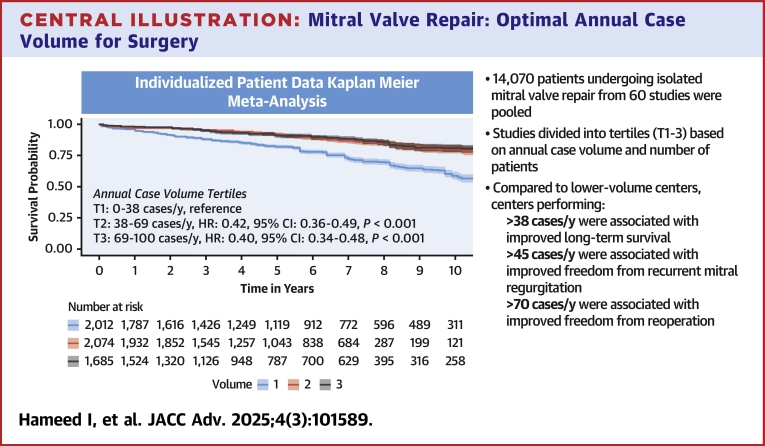
Mitral Valve Repair: Optimal Annual Case Volume for Surgery Long-term survival for patients undergoing isolated mitral valve repair stratified by volume tertiles (T1-T3).

#### Long-term freedom from reoperation

**Figure 1:**
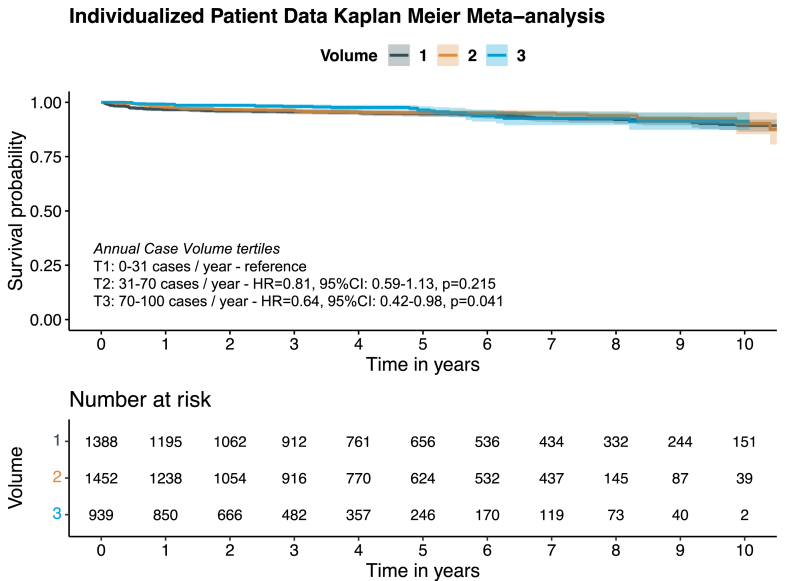
Long-Term Freedom From Reoperation for Patients Undergoing Isolated Mitral Valve Repair Stratified by Volume Tertiles (T1-T3)

Twelve studies (n = 4,230) reported long-term freedom from reoperation. The overall pooled 10-year freedom from reoperation was 90.2% (95% CI: 88.1%-92.4%). Centers were divided into the following individual volume tertiles for assessment: T1 = 0 to 31 cases/y, T2 = 31 to 70 cases/y, and T3 = 70 to 100 cases/y. The highest tertile centers were associated with significantly better long-term freedom from reoperation compared to the lowest tertile centers (HR: 0.64; 95% CI: 0.42-0.98; *P* = 0.042) (test for proportionality of hazard; *P* < 0.01) (Figure 1). Additional analysis using RMST showed similar results with T3 having a significantly better long-term freedom from reoperation compared to T1 (*P* < 0.01). However, in a subgroup analysis of 5 studies (919 patients) reporting long-term freedom from reoperation following isolated MVr in patients with degenerative valve disease, there was no significant volume/outcome association (T3 vs T1: HR: 1.36; 95% CI: 0.45-4.11; *P* = 0.58) (test for proportionality of hazard; *P* < 0.01) (Supplemental Figure 3). Again, similar results were observed in an analysis using RMST.

#### Long-term freedom from recurrent mitral regurgitation

**Figure 2:**
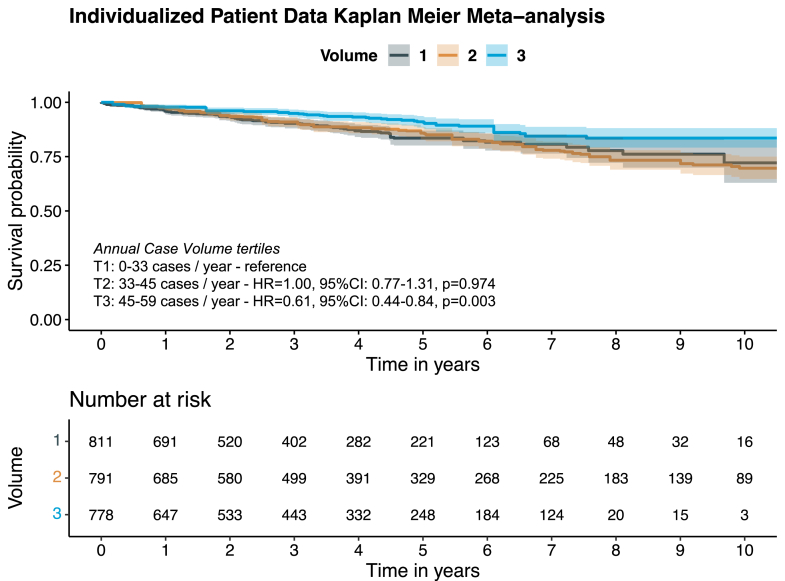
Long-Term Freedom From Recurrent Mitral Regurgitation (Moderate-Severe) for Patients Undergoing Isolated Mitral Valve Repair Stratified by Volume Tertiles (T1-T3)

Ten studies (n = 2,470) reported Kaplan-Meier derived long-term freedom from recurrent mitral regurgitation data. The overall pooled 10-year freedom from recurrent mitral regurgitation was 72.7% (95% CI: 68.9%-76.8%). Based on their number of patients, centers were grouped into the following individual volume tertiles: T1 = 0 to 33 cases/y, T2 = 33 to 45 cases/y, and T3 = 45 to 59 cases/y. Compared to the lowest tertile centers, the highest tertile centers were associated with significantly improved long-term freedom from recurrent mitral regurgitation (T3 vs T1: HR: 0.61; 95% CI: 0.44-0.84; *P* = 0.003) (test for proportionality of hazard; *P* = 0.13) (Figure 2). This was true in a subgroup analysis limited to patients with degenerative mitral disease pooling 8 studies with 2,146 patients (T3 vs T1: HR: 0.51; 95% CI: 0.36-0.72; *P* < 0.001) (test for proportionality of hazard; *P* = 0.06) (Supplemental Figure 4).

### Secondary outcomes

#### Early (postoperative/in-hospital/30-day) mortality

**Figure 3:**
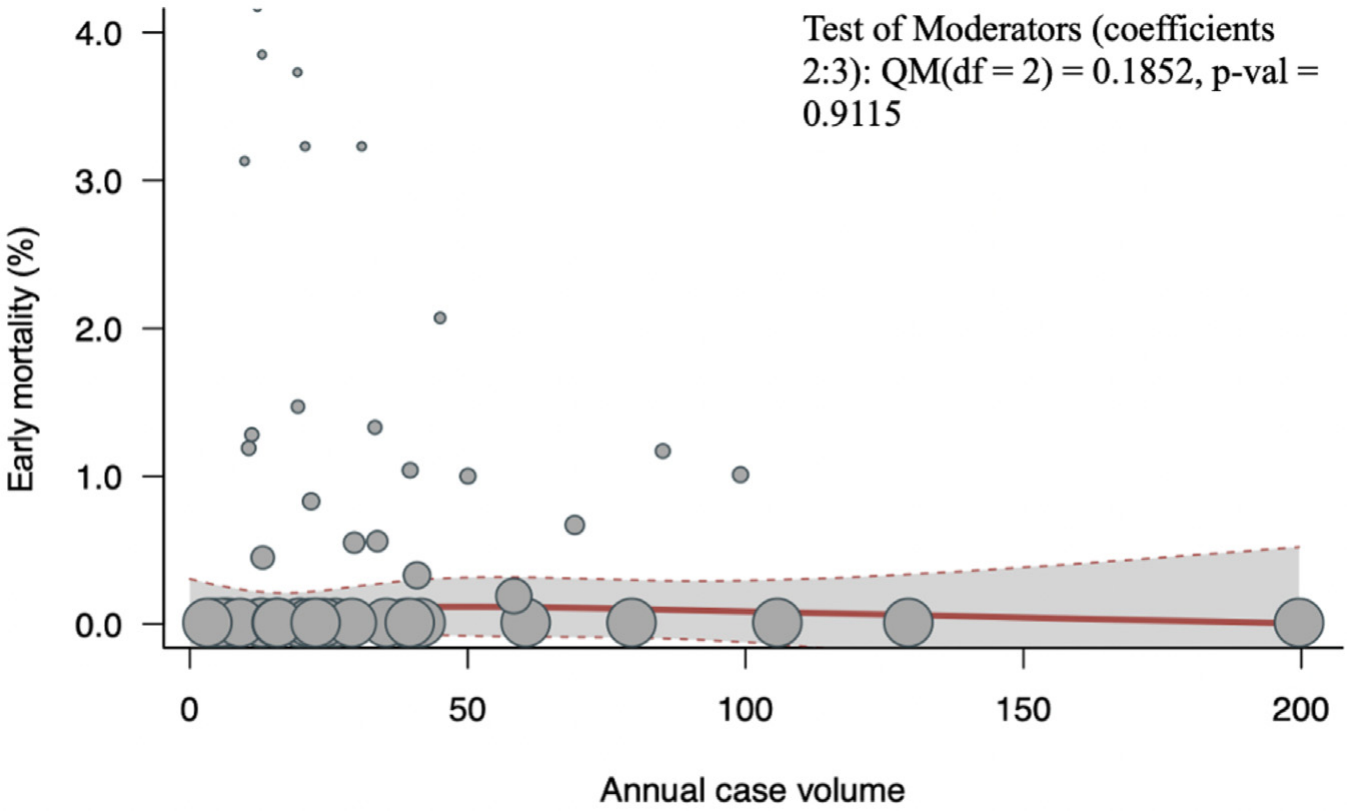
Volume-Outcome Association for Early Mortality

Early mortality was reported by 53 studies (n = 11,965). The overall pooled early mortality (postoperative/in-hospital/30-day) was 1.14% (95% CI: 0.81%-1.62%; I^2^ = 65.2%; *P* < 0.001). There was no significant association between volume and early mortality (*P* = 0.91) (Figure 3). Similarly, in a subgroup analysis of patients with degenerative mitral valve disease, there was no significant association between volume and early mortality (*P* = 0.93) (Supplemental Figure 5).

#### Early (postoperative/in-hospital/30-day) stroke

**Figure 4:**
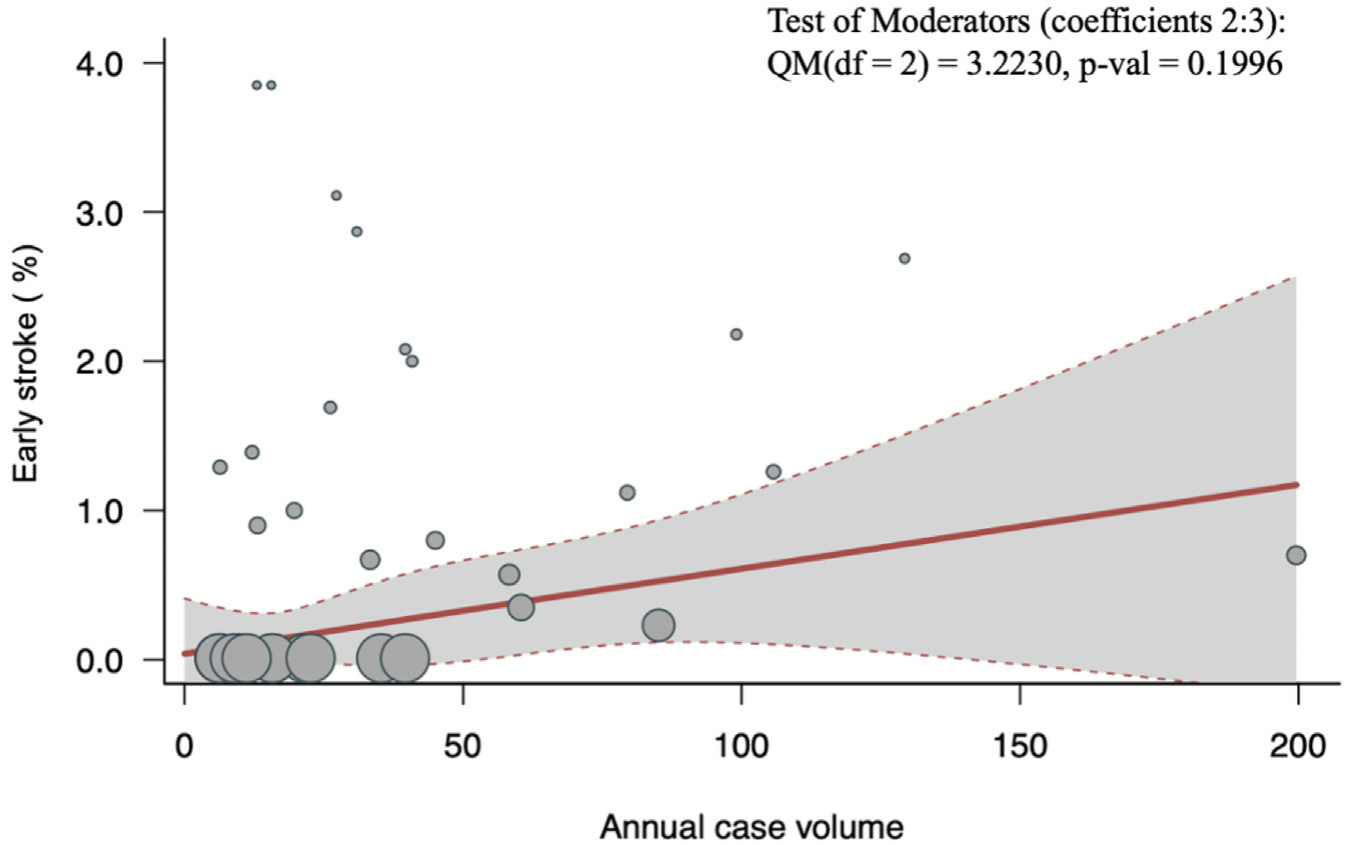
Volume-Outcome Association for Early Stroke

Thirty-two studies (n = 7,317) reported early stroke in their series. The overall pooled early (postoperative/in-hospital/30-day) stroke was 1.46% (95% CI: 1.10-1.95; I^2^ = 30.9%; *P* = 0.047). There was no significant association between volume and early stroke (*P* = 0.20) (Figure 4). We observed similar results in a subgroup analysis of patients with degenerative mitral valve disease (*P* = 0.54) (Supplemental Figure 6).

#### Early (postoperative/in-hospital/30-day) major bleeding

**Figure 5:**
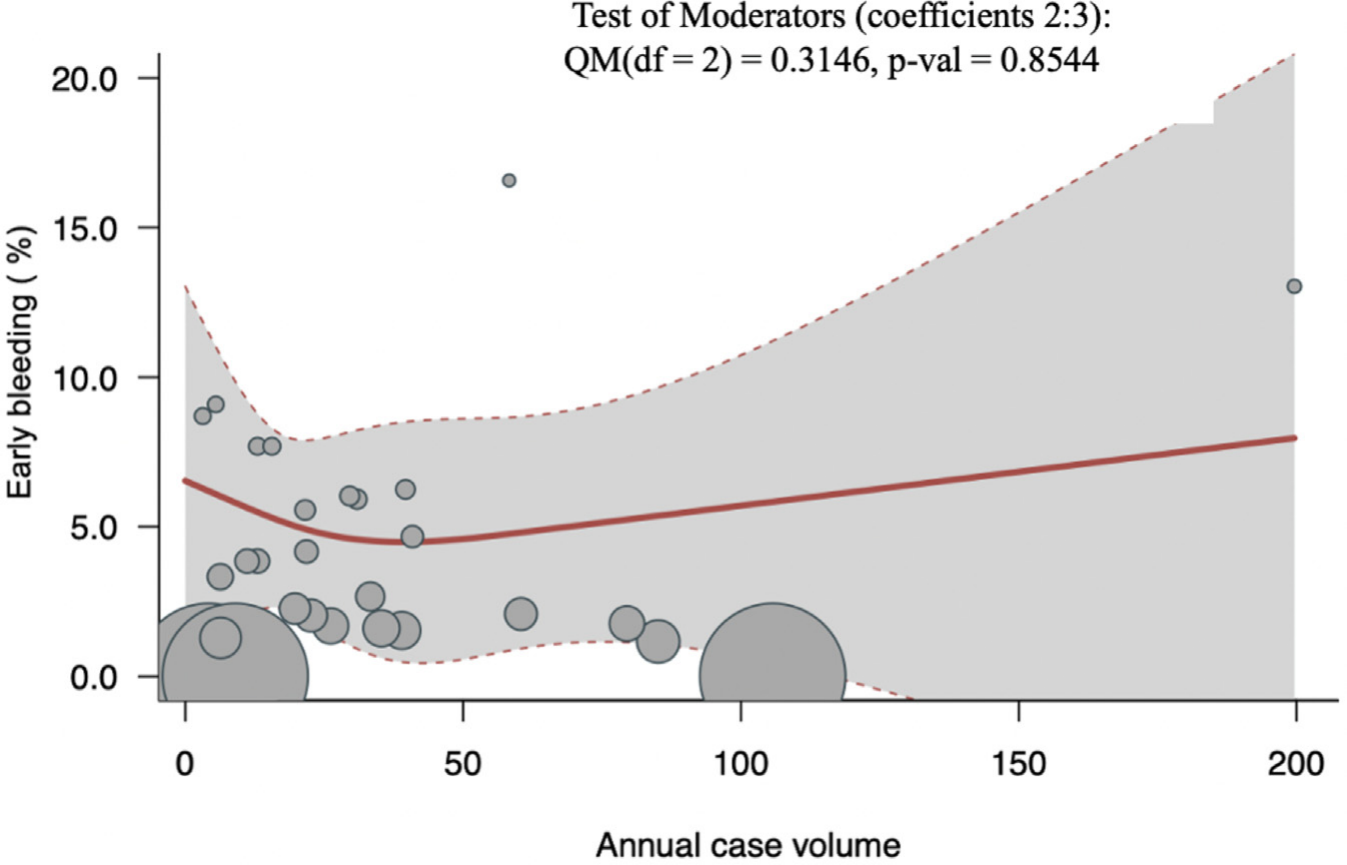
Volume-Outcome Association for Early Major Bleeding

Early (postoperative/in-hospital/30-day) major bleeding was reported by 29 studies (6,212 patients). Overall pooled early major bleeding was 4.26% (95% CI: 2.88-6.26; I^2^ = 89.9%; *P* < 0.001). There was no significant association between volume and early major bleeding (*P* = 0.85) (Figure 5). For patients with degenerative mitral valve disease, however, there was significant association between volume and early major bleeding (*P* = 0.02) (Supplemental Figure 7).

#### Early (postoperative/in-hospital/30-day) renal failure

**Figure 6:**
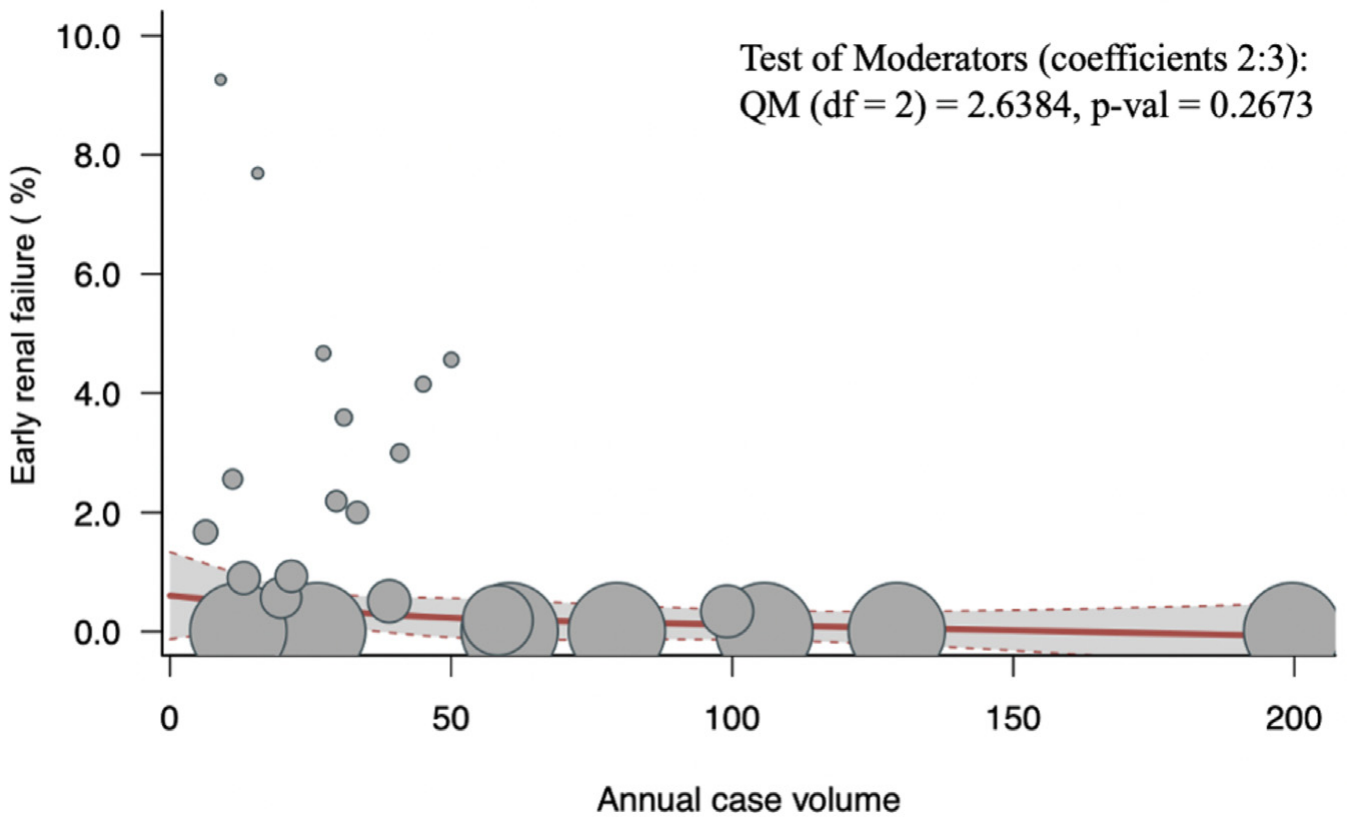
Volume-Outcome Association for Early Renal Failure

Twenty-four studies (n = 7,874) reported data on early (postoperative/in-hospital/30-day) renal failure. The overall pooled renal failure was 1.45% (95% CI: 0.85-2.44; I^2^ = 71.8%; *P* < 0.001). There was no significant association between volume and early renal failure (*P* = 0.27) (Figure 6). Similarly, in a subgroup analysis of patients with degenerative mitral valve disease, there was no significant association between volume and early renal failure (*P* = 0.81) (Supplemental Figure 8).

##### Publication bias

Significant publication bias was observed for early mortality (*P* < 0.001), early stroke (*P* = 0.032), early major bleeding (*P* < 0.001), and early renal failure (*P* < 0.001), suggesting underreporting of studies with higher incidence of these complications (Supplemental Figure 9).

## Discussion

This analysis of 60 studies from 58 centers worldwide evaluates the association between isolated MVr volume and contemporary long-term patient outcomes. By pooling individual patient data from 14,070 patients undergoing isolated MVr, we identify important volume cutoffs to distinguish mitral centers of excellence and guide referrals. We found MVr volume of >38 cases/y to be associated with significantly improved survival at 10 years. Improvement in long-term durability of repair and freedom from reoperation, however, was associated with higher MVr volumes of >45 cases/y and >70 cases/y, respectively. These findings were largely true for analyses limited to patients undergoing repair for degenerative mitral valve disease only. Our tiered analysis of centers by volume tertiles further confirmed improved survival, durability of repair, and freedom from reoperation with increasing volume. We also evaluated association of center volumes with short-term outcomes. While there was a trend toward improvement of outcomes with higher volume, this was not statistically significant, presumably due to the low incidence of symptomatic complications in the early postoperative period following repair.^20^

It is currently estimated that ∼10% of patients undergoing MVr will require reoperation during their lifetime.^21^^,^^22^ The indications for reoperation often include valve-related complications, such as progression of degenerative valve disease or the development of new lesions, which can occur irrespective of the quality of initial repair.^23^ Alarmingly, the rate of mitral valve reoperation related to technical repair failures, such as suture/ring dehiscence, neochord complications, and left ventricular pseudoaneurysms is as high as 41%.^21^ Several features of high-volume centers explain their improved outcomes following MVr. Such centers have better practices for guiding patient selection and more experience in the postoperative management of patients undergoing MVr. Furthermore, experienced surgeons in such centers can mentor less experienced colleagues in patient selection and guide preoperative and intraoperative planning and repair strategy. Such centers of excellence are also characterized by high-quality multidisciplinary collaboration between interventional cardiology, structural cardiology imaging, and cardiac surgery which streamlines preoperative evaluation, risk stratification, and surveillance of patients.^6^

To date, some regional and national database studies have assessed the relationship between annual mitral valve surgery volume, repair rates, and outcomes. In a 2017 New York State registry study of adult patients undergoing primary MVr, Chikwe et al^6^ found increased annual surgeon volume to be significantly associated with higher rate of successful repairs (adjusted OR: 1.13/10 additional mitral valve surgeries; 95% CI: 1.10-1.17; *P* < 0.001) and superior 1-year survival (adjusted HR 0.95/10 additional mitral valve surgeries; 95% CI 0.92-0.98; *P* = 0.001). Another report published in 2020 by Badhwar and colleagues assessed 30-day and 1-year outcomes in patients undergoing isolated MVr or replacement in the Society of Thoracic Surgeons Adult Cardiac Surgery Database.^5^ They found centers in the lowest volume quartile to less frequently attempt MVr compared to the highest volume quartile (63.8% vs 84.5%; *P* < 0.001) and have lower rates of successful MVr (adjusted OR: 0.50; 95% CI: 0.34-0.72; *P* < 0.001). Moreover, centers from the lowest volume quartile were associated with a significantly higher 1-year mortality (HR: 1.61; 95% CI: 1.31-1.98). There was, however, no statistically significant difference in the rate of reoperation at 1 year (OR: 1.51; 95% CI: 0.81-2.78) between the lowest and highest volume quartile centers. We found a high threshold of >70 cases/y to confer significant freedom from reoperation at 10 years. While important, prior reports have limitations of administrative databases which do not accurately distinguish different disease etiologies, potentially erroneous coding, and have limited follow-up limited to 1 year. Additionally, the data sets were limited to specific geographic regions limiting their generalizability. For this analysis, we applied a novel meta-analytical volume-outcome approach previously described by our group.^10^ By applying this approach, we have consolidated over a decades-worth of real-word international MVr data without geographical restrictions. Additionally, our threshold for volumes associated with improved long-term survival was determined from granular Kaplan-Meier-derived individual patient data. This minimizes the risk of selection bias encountered in registry-sourced studies where patients may be lost to follow-up.

Our data support a volume threshold of 70 cases/y for MVr referral centers to confer maximum benefit in terms of long-term survival, durability of repair, and freedom from reoperation. This proposition is higher than the arbitrary 50 cases/y cutoff recommended by Bridgewater et al^8^ about 2 decades ago which is often used to guide recommendations. More recently in 2022, Wayne et al,^9^ using data from the Australian and New Zealand Society of Cardiac and Thoracic Surgeons registry, proposed a minimum of 10 mitral valve operations per year as cutoff. They found hospitals under this threshold to have significantly higher rates of repair attempts (10.1-20 cases/y; OR: 1.96; 95% CI: 1.25-3.07; *P* = 0.003). Consistent with our analysis, the authors did not find an association between higher volume and lower 30-day mortality (0.96%) (OR: 0.55; 95% CI: 0.32-0.93; *P* = 0.28). Of note, the pooled early mortality of 1.14% in our analysis is comparable to the 30-day isolated MVr mortality incidence (1.16%) reported in the Society of Thoracic Surgeons Adult Cardiac Surgery Database.^20^

### Study Limitations

For relevance to contemporary practice, we limited our analysis to studies published from 2013. This potentially limited our ability to calculate individual operator thresholds and include unreported variables in our regression analyses. Moreover, the pooling of nonrandomized, single-center studies predispose us to variability in cohort sample size, risk factors, and peri-operative/postoperative management protocols. Accordingly, our analysis for early mortality, major bleeding, and renal failure showed high heterogeneity between the studies, which may limit the generalizability of those findings. Similarly, the use of a meta-analytical approach risks publication bias as referral centers of excellence are more likely to publish their positive outcomes than less experienced centers with higher complications. In particular, we found evidence suggesting publication bias in our analyses for early mortality, stroke, major bleeding and renal failure, and readers must carefully interpret our findings in this context. Our pooled outcomes, however, are consistent with those reported in large registry studies and likely representative of general practice. Additionally, although we were able to extrapolate individual patient-level long-term outcome data from the digitization of the published curves, we were unable to adjust for variables in our analysis due to inclusion of study-level data only and lack of access to individual patient data, surgical variables, and baseline comorbidities. We also excluded multicenter studies as we are unable to accurately determine annual repair case volumes of the individual centers. Also, studies including patients undergoing concomitant operations with MVr were excluded. Our analyses were limited to institutional case volumes, and we were unable to obtain individual surgeon case volume data. Unlike very complex patients where overall hospital caseload affects outcomes due to involvement of the entire peri-operative/postoperative program, in isolated MVr patients, it is probable that individual surgeons and techniques influence outcomes. Thus, a “Valve Center of Excellence” is as much defined by its individual surgical expertise as its overall caseload.

## Conclusions

In summary, we describe specific volume cutoffs for optimal long-term outcomes following isolated MVr. MVr volumes of >38 cases/y were associated with significantly longer survival at 10 years follow-up. Higher MVr volumes of 45 cases/y and >70 cases/y were associated with significantly improved durability of repair and freedom from reoperation, respectively, at 10 years. Our findings are important for defining experienced centers and surgeons for patients requiring MVr.

## Funding support and author disclosures

The authors have reported that they have no relationships relevant to the contents of this paper to disclose.

## Supplementary data

Supplemental Figures 1, 2, 3, 4, 5, 6, 7, 8, and 9, and Supplemental Tables 1, 2, 3, 4, 5, 6, and 7
