# Supplementary material for: Mitral Valve Repair: Optimal Annual Case Volume for Surgery
Source: JACC Adv. 2025 Feb 10;4(3):101589. doi: 10.1016/j.jacadv.2025.101589 (PMC11867518; doi:10.1016/j.jacadv.2025.101589)
Supplement: Supplemental Figures 1, 2, 3, 4, 5, 6, 7, 8, and 9, and Supplemental Tables 1, 2, 3, 4, 5, 6, and 7 [file mmc1.docx]

**Supplemental Figure 1.** Preferred Reporting Items for Systematic Reviews and Meta-Analysis (PRISMA) flowchart of our analysis.

I**dentification**

Additional records from through other sources
(n = 13)

Records identified from database searching (n= 21,183)

Full text articles excluded, with reason (n=2560)

- NonEnglish (n=56)
- Case report (n=248)
- Duplicate (n=1)
- Studies irrelevant to the study question (n=1012)
- Systematic reviews/meta-analyses (n=222)
- Animal studies/in vitro (n=49)
- Editorials (n=230)
- Overlapping patient cohorts (n=64)
- Concomitant procedures / mitral valve replacement (n=518)
- Mitral valve reoperation (n=9)
- Multicenter cohorts (n=83)
- Pediatric population (n=32)
- Percutaneous mitral valve repair (n=36)

**Eligibility**

**Included**

Studies included in quantitative synthesis (meta-analysis)

(n = 60)

Studies included in qualitative synthesis

(n=60)

**Screening**

Records excluded based on nonpertinent title/abstract

(n = 18,268)

Records screened
(n = 2,620)

Records after duplicates removed

(n=20,888)

Full-text articles assessed for eligibility
(n = 2,620)

**Supplemental Figure 2.** Long-term survival for patients with degenerative disease undergoing isolated mitral valve repair, stratified by volume tertiles (T1-T3). CI: Confidence interval; HR: Hazard ratio.

**Supplemental Figure 3.** Long-term freedom from re-operation for patients with degenerative disease undergoing isolated mitral valve repair.

**Supplemental Figure 4.** Long-term from >mild mitral regurgitation recurrence for patients with degenerative disease undergoing isolated mitral valve repair.

**Supplemental Figure 5.** Volume-outcome association for early mortality in patients with degenerative mitral valve disease.

Test of Moderators (coefficients 2:3): QM (df = 2) = 0.1540, p-val = 0.9259


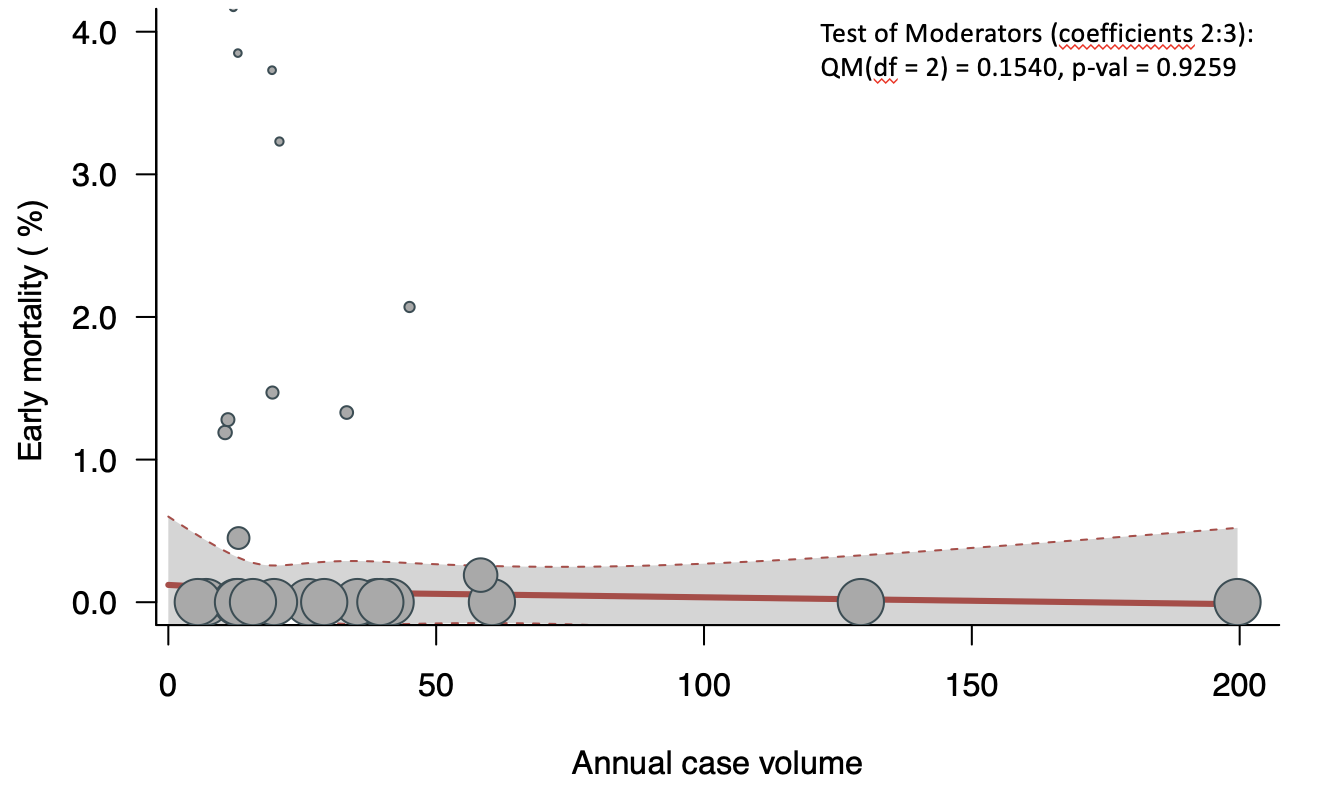


**Supplemental Figure 6.** Volume-outcome association for early stroke in patients with degenerative mitral valve disease.


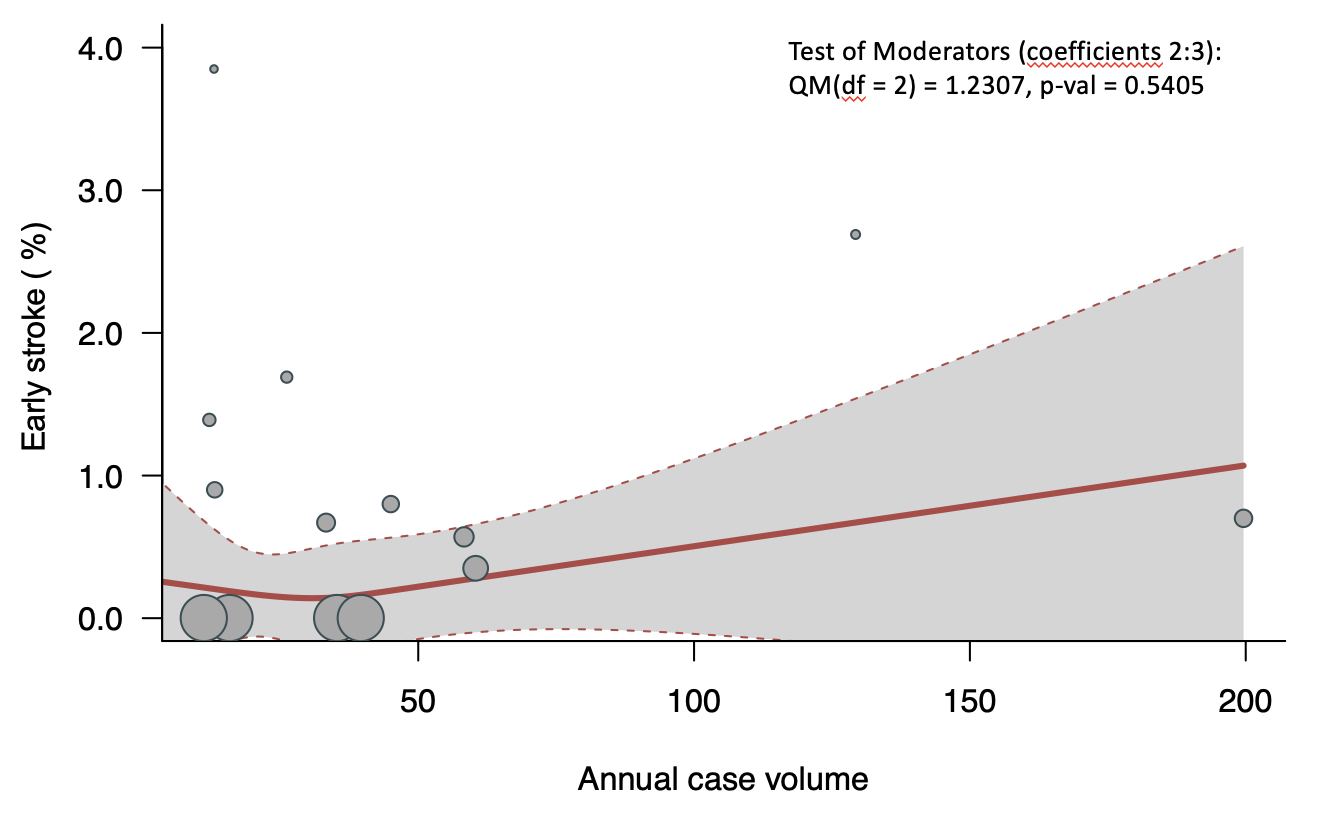


Test of Moderators (coefficients 2:3): QM(df = 2) = 1.2307, p-val = 0.5405

**Supplemental Figure 7.** Volume-outcome association for early major bleeding in patients with degenerative mitral valve disease.


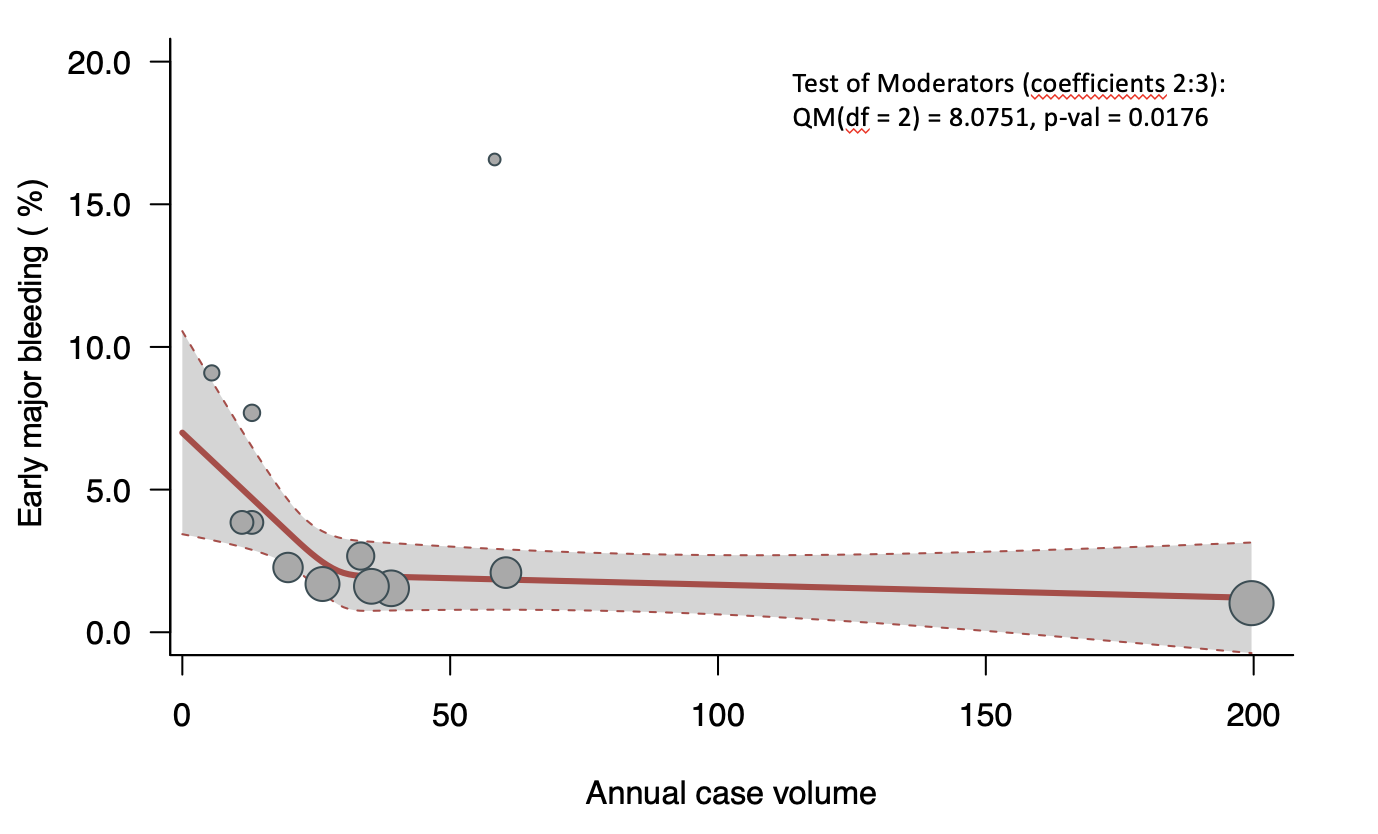


Test of Moderators (coefficients 2:3): QM(df = 2) = 8.0751, p-val = 0.0176

**Supplemental Figure 8.** Volume-outcome association for early renal failure in patients with degenerative mitral valve disease.


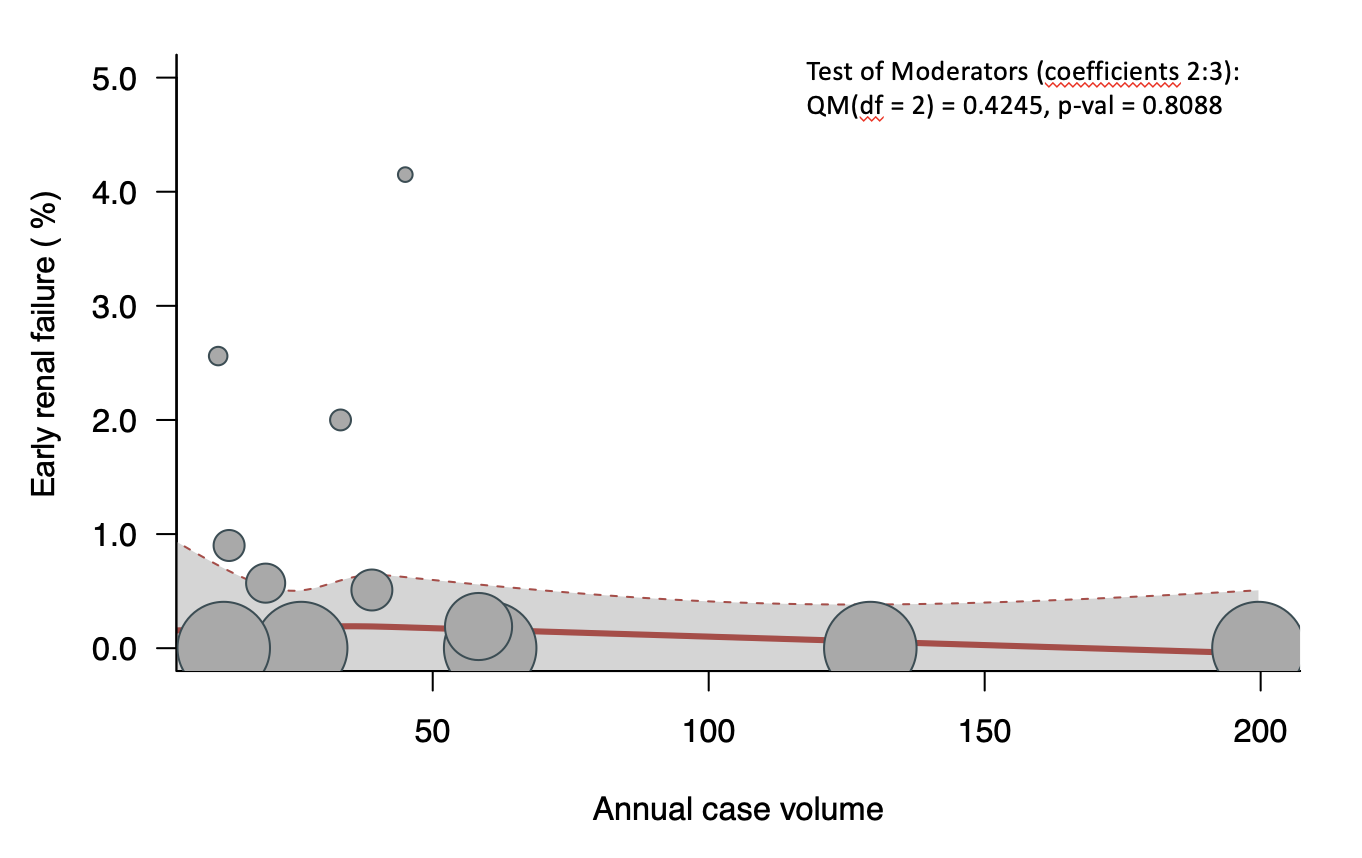


Test of Moderators (coefficients 2:3): QM(df = 2) = 0.4245, p-val = 0.8088

**Supplemental Figure 9.** Funnel plots assessing publication bias for short-term outcomes.

**
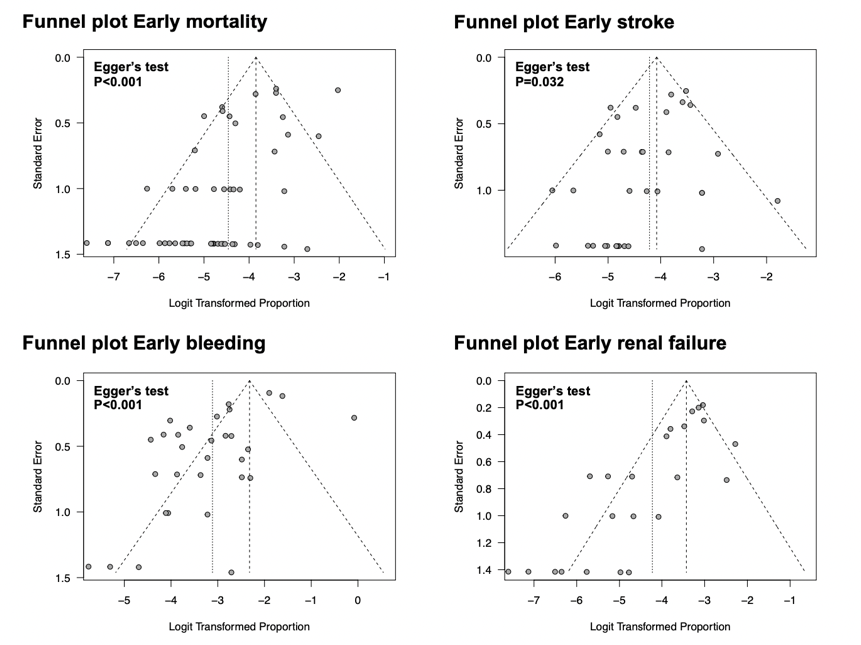
**

**Supplemental Table 1.** Detailed search strategy.

November 14, 2023

Database: Ovid MEDLINE(R) ALL <1946 to November 14, 2023>

1 (mitral valve adj2 (repair* or surger*)).tw,kw,kf. 10245

2 Mitral Valve/su [Surgery] 16541

3 Mitral Valve Insufficiency/su [Surgery] 13012

4 Mitral Valve Stenosis/su [Surgery] 6056

5 Mitral Valve Prolapse/su [Surgery] 1076

6 1 or 2 or 3 or 4 or 5 30697

7 exp treatment outcome/ 1258539

8 ((treatment or clinical or hospital or patient* or rehabilitation) adj1 (outcome* or effectiveness or efficacy)).tw,kw,kf. 489838

9 exp Outcome Assessment, Health Care/ 1355053

10 (outcome* adj2 (assessment* or measure* or study or studies)).mp. 476174

11 echocardiograph* outcome*.tw,kw,kf. 446

12 exp Echocardiography/mt, sn [Methods, Statistics & Numerical Data] 32378

13 7 or 8 or 9 or 10 or 11 or 12 1998511

14 6 and 13 9356

15* 14 not ((exp infant/ or exp child/ or adolescent/) not exp adult/) 8876

16 limit 15 to yr="2012 2023" 5877 citations yielded

Embase <1974 to 2023 November 17>

1 (mitral valve adj2 (repair* or surger*)).tw,kw,kf. 15534

2 Mitral Valve/su [Surgery] 2514

3 Mitral Valve regurgitation/su [Surgery] 11786

4 Mitral Valve Stenosis/su [Surgery] 4507

5 Mitral Valve Prolapse/su [Surgery] 1228

6 1 or 2 or 3 or 4 or 5 28017

7 exp treatment outcome/ 2328727

8 ((treatment or clinical or hospital or patient* or rehabilitation) adj1 (outcome* or effectiveness or efficacy)).tw,kw,kf. 776904

9 exp Outcome Assessment/ 904594

10 (outcome* adj2 (assessment* or measure* or study or studies)).mp. 1273924

11 echocardiogra* outcome*.tw,kw,kf. 880

12 exp echocardiography/ and outcome$1.tw,kw,kf. 55793

13 or/7-12 3034184

14 6 and 13 8372

15 14 not ((exp infant/ or exp child/ or adolescent/) not exp adult/) 7993

16 limit 15 to yr="2012 - 2023" 5518

Cochrane CENTRAL, 357 results

ID Search Hits

#1 mitral valve NEAR/2 (repair* or surger*) 920

#2 (treatment or clinical or hospital or patient* or rehabilitation) NEAR/1 (outcome* or effectiveness or efficacy) 311769

#3 outcome* NEAR/2 (assessment* or measure* or study or studies) 222443

#4 echocardiogra* outcome* 8112

#5 #2 OR #3 OR #4 453793

#6 #1 AND #5 with Publication Year from 2012 to 2023, in Trials 357

**Supplemental Table 2.** Demographics of included patients.

| Study/Year | No Patients | Mean Age | SD Age | % Female | Mean EuroScore | ED EuroScore | Mean EuroScore II | SD EuroScore II |
| --- | --- | --- | --- | --- | --- | --- | --- | --- |
| Afshar, 2023 | 75 | 59.7 | 8.9 | 34.7 | NR | NR | NR | NR |
| Agnino, 2022 | 59 | 58 | 12 | 10.2 | NR | NR | 0.93 | 0.5 |
| Agnino, 2019 | 37 | 62.6 | 13.4 | 35 | NR | NR | 1.3 | 0.8 |
| Ascaso, 2023 | 300 | 61 | 12 | 32 | NR | NR | NR | NR |
| Atluri, 2016 | 159 | 62 | NR | 39 | NR | NR | NR | NR |
| Balachandran, 2020 | 720 | 61.1 | 52.7-69.1 | 27.6 | NR | NR | NR | NR |
| Bellitti, 2014 | 140 | 56.4 | 14.5 | 27.9 | NR | NR | NR | NR |
| Berdajs, 2023 | 365 | 61 | 11 | 36 | 2.7 | 1.8-4.8 | NR | NR |
| Chemtob, 2022 | 998 | 58 | 11 | 23 | NR | NR | NR | NR |
| Chi, 2014 | 12 | NR | NR | NR | NR | NR | NR | NR |
| D’Onofrio, 2022 | 176 | 61.5 | 53.5-70.7 | 25 | NR | NR | 0.78 | 0.55-1 |
| El-Andari, 2021 | 701 | 63.3 | 13.2 | 28.5 | NR | NR | NR | NR |
| Franke, 2022 | 96 | 58.8 | 11.1 | 21.9 | NR | NR | 1.4 | 1.2 |
| Garatti, 2018 | 134 | NR | NR | NR | NR | NR | NR | NR |
| Garcia Fuster, 2014 | 68 | 64 | 11.4 | 33.8 | 4.5 | 2.2 | NR | NR |
| Gerber, 2019 | 114 | 49.4 | 14.4 | 31.6 | NR | NR | NR | NR |
| Goldstone, 2015 | 525 | 57.6 | 12.4 | 31.6 | NR | NR | NR | NR |
| Götte, 2022 | 257 | 78 | 76-80 | 53.7 | 11.2 | 7-16.6 | NR | NR |
| Grapsa, 2015 | 64 | 63.6 | 58.6-76.9 | 48 | 5.37 | 2.6 | NR | NR |
| Grinberg, 2019 | 7 | 80 | 63-82 | NR | NR | NR | 1.4 | 1.1-2.8 |
| Güllü, 2021 | 60 | 48.3 | NR | 61.7 | NR | NR | NR | NR |
| Hasham, 2022 | 287 | 62.1 | 11.8 | 40 | NR | NR | NR | NR |
| Hayashi, 2021 | 100 | 63.3 | 15.2 | 40 | NR | NR | 1.8 | 2.1 |
| Hu, 2021 | 623 | 54.8 | 12.1 | 41.4 | NR | NR | NR | NR |
| Huang, 2013 | 48 | 47 | 12-68 | 27.1 | NR | NR | NR | NR |
| Javadikasgari, 2017 | 623 | 56.2 | 9.7 | 15 | NR | NR | NR | NR |
| Kamiya, 2017 | 22 | 51.6 | 17.2 | 45.5 | NR | NR | 0.99 | 0.6 |
| Kitai, 2014 | 473 | 57 | 14 | 45 | NR | NR | NR | NR |
| Kurlansky, 2023 | 434 | 72.5 | 4.9 | 52.3 | NR | NR | NR | NR |
| Lange, 2017 | 745 | 57.9 | 13 | 32 | NR | NR | NR | NR |
| Lee, 2018 | 38 | 42.3 | 18.8 | 36.8 | NR | NR | NR | NR |
| Levy, 2022 | 39 | 60 | 14 | 50 | NR | NR | 2.1 | 1.6 |
| Li, 2022 | 120 | 49.91 | 12.12 | 35 | NR | NR | NR | NR |
| Lio, 2017 | 25 | 56 | 14 | NR | NR | NR | NR | NR |
| Ma, 2019 | 390 | 52.7 | 12.2 | 35.6 | NR | NR | NR | NR |
| Magruder, 2016 | 223 | 59 | 13 | 37.7 | NR | NR | NR | NR |
| Makarious, 2023 | 64 | 70.5 | 42-86 | 46.9 | NR | NR | 1.5 | 0.56-14.4 |
| Mihaljevic, 2013 | 334 | 56.3 | 9.7 | 20.3 | NR | NR | NR | NR |
| Moscarelli, 2018 | 62 | 64.7 | 11.9 | 54.9 | 3.7 | 5 | NR | NR |
| Moscarelli, 2021 | 54 | 68 | 8.5 | 43 | NR | NR | 4.04 | 2.3 |
| Muneretto, 2015 | 50 | 53 | 11 | 38 | 3.1 | 1.4 | NR | NR |
| Murzi, 2014 | 595 | 60.8 | 13.2 | 44.9 | 4.6 | 3.2 | NR | NR |
| Noack, 2019 | 557 | 62.8 | 11.7 | 42.6 | 7.2 | 5.7 | NR | NR |
| Panos, 2015 | 426 | 55 | 18 | NR | NR | NR | NR | NR |
| Paranskaya, 2013 | 26 | 63 | 12 | 34.6 | 3.9 | 3.7 | NR | NR |
| Perin, 2019 | 108 | 60.5 | 12.5 | 23.1 | 4.21 | 3.48 | NR | NR |
| Petolat, 2023 | 198 | 64 | 13 | 51 | NR | NR | NR | NR |
| Ramzy, 2014 | 300 | 57.3 | 11 | 36 | NR | NR | NR | NR |
| Salihi, 2019 | 26 | 56 | 10.1 | 30.8 | NR | NR | 1.04 | 0.7 |
| Shibata, 2015 | 180 | 64.1 | 12.1 | 38 | NR | NR | NR | NR |
| Smith, 2016 | 63 | 57.7 | 10.4 | NR | NR | NR | NR | NR |
| Solari, 2019 | 155 | 60.1 | 13.8 | 30.6 | NR | NR | NR | NR |
| Tomšič, 2019 | 627 | 67.1 | 11.78 | 37 | NR | NR | 1.89 | 1.97 |
| Uchimuro, 2014 | 119 | 58.4 | 11.8 | 31.9 | NR | NR | NR | NR |
| Vairo, 2023 | 72 | 77 | 9 | 37.4 | NR | NR | 2.2 | 1.5 |
| Van Leeuwen, 2013 | 46 | 50.2 | 13.2 | 11 | NR | NR | NR | NR |
| Van Praet, 2022 | 491 | 62.4 | 13.6 | 37.9 | NR | NR | 1.2 | 2.8 |
| Wang, 2016 | 84 | 47.8 | 11.9 | 28.6 | NR | NR | NR | NR |
| Westhofen, 2016 | 98 | 56.5 | 11.8 | 38 | NR | NR | 0.84 | 0.39 |
| Zorinas, 2019 | 78 | 59.5 | 12.8 | 32.1 | NR | NR | 0.83 | 0.67-1.35 |
|  |  |  |  |  |  |  |  |  |
| Study/Year | **Mean STS Score** | **ED STS Score** | **% NYHA III/IV** | **Mean BMI** | **SD BMI** | **Mean BSA** | **SD BSA** | **% HTN** |
| Afshar, 2023 | NR | NR | NR | 26.9 | 4.72 | NR | NR | 60 |
| Agnino, 2022 | NR | NR | 27.1 | NR | NR | 1.9 | 0.2 | NR |
| Agnino, 2019 | 0.8 | NR | 54.1 | NR | NR | NR | NR | NR |
| Ascaso, 2023 | NR | NR | 33.5 | 25.7 | 4 | NR | NR | 42 |
| Atluri, 2016 | 0.53 | NR | NR | NR | NR | NR | NR | NR |
| Balachandran, 2020 | NR | NR | NR | 25.9 | 23.4-28.7 | 2 | 1.8-2.1 | 47.1 |
| Bellitti, 2014 | NR | NR | 43.6 | NR | NR | NR | NR | NR |
| Berdajs, 2023 | NR | NR | 26 | 25 | 4.2 | NR | NR | 58 |
| Chemtob, 2022 | NR | NR | 2.6 | 26 | 4.2 | NR | NR | 46.6 |
| Chi, 2014 | NR | NR | NR | NR | NR | NR | NR | NR |
| D’Onofrio, 2022 | NR | NR | 17 | NR | NR | 1.8 | 1.7-1.9 | 45 |
| El-Andari, 2021 | 0.94 | 0.82 | NR | NR | NR | NR | NR | 60.5 |
| Franke, 2022 | NR | NR | NR | NR | NR | NR | NR | NR |
| Garatti, 2018 | NR | NR | NR | NR | NR | NR | NR | NR |
| Garcia Fuster, 2014 | NR | NR | 54.4 | NR | NR | NR | NR | 61.8 |
| Gerber, 2019 | NR | NR | NR | NR | NR | NR | NR | NR |
| Goldstone, 2015 | NR | NR | 68.4 | 26.4 | 7.1 | NR | NR | 43.6 |
| Götte, 2022 | NR | NR | 52.5 | 25.1 | 23.5-28 | NR | NR | NR |
| Grapsa, 2015 | NR | NR | NR | 26.7 | 22-29.5 | NR | NR | NR |
| Grinberg, 2019 | NR | NR | 71 | NR | NR | NR | NR | NR |
| Güllü, 2021 | NR | NR | 10 | NR | NR | NR | NR | 10 |
| Hasham, 2022 | 0.63 | 0.66 | NR | 26 | 3.9 | 1.91 | 0.27 | 44 |
| Hayashi, 2021 | NR | NR | NR | NR | NR | 1.59 | 0.21 | 51 |
| Hu, 2021 | NR | NR | NR | NR | NR | 1.75 | 0.28 | 11.4 |
| Huang, 2013 | NR | NR | NR | NR | NR | NR | NR | NR |
| Javadikasgari, 2017 | NR | NR | 6.18 | NR | NR | NR | NR | 45 |
| Kamiya, 2017 | NR | NR | 36.3 | 23.5 | 3.6 | NR | NR | 40.9 |
| Kitai, 2014 | NR | NR | NR | NR | NR | 1.59 | 0.19 | NR |
| Kurlansky, 2023 | NR | NR | 68.2 | NR | NR | NR | NR | 51.4 |
| Lange, 2017 | NR | NR | NR | NR | NR | NR | NR | NR |
| Lee, 2018 | NR | NR | NR | NR | NR | NR | NR | NR |
| Levy, 2022 | NR | NR | 31 | NR | NR | NR | NR | 31 |
| Li, 2022 | NR | NR | NR | 24.84 | 5.59 | NR | NR | 30.8 |
| Lio, 2017 | NR | NR | NR | NR | NR | NR | NR | NR |
| Ma, 2019 | NR | NR | 37.9 | NR | NR | 1.67 | 0.23 | 29.5 |
| Magruder, 2016 | 0.9 | 0.2 | NR | 26 | 5 | NR | NR | 42.6 |
| Makarious, 2023 | NR | NR | 73.4 | NR | NR | NR | NR | 79.7 |
| Mihaljevic, 2013 | NR | NR | 8.4 | 25.8 | 4.1 | NR | NR | 41 |
| Moscarelli, 2018 | NR | NR | 16.1 | 25.2 | 3.4 | NR | NR | 19.3 |
| Moscarelli, 2021 | NR | NR | 100 | 26.8 | 4.4 | NR | NR | 61 |
| Muneretto, 2015 | NR | NR | 84 | 23.9 | 4.3 | NR | NR | 48 |
| Murzi, 2014 | NR | NR | NR | 27.8 | 5 | NR | NR | 51.8 |
| Noack, 2019 | NR | NR | 55.1 | 26.8 | 5.5 | NR | NR | 64 |
| Panos, 2015 | NR | NR | NR | NR | NR | NR | NR | NR |
| Paranskaya, 2013 | 1.4 | 1.6 | 96.2 | 27 | 4 | NR | NR | 57.7 |
| Perin, 2019 | NR | NR | 23.1 | 26 | 3.66 | NR | NR | 28.7 |
| Petolat, 2023 | NR | NR | 22 | NR | NR | NR | NR | 23 |
| Ramzy, 2014 | NR | NR | 43.3 | 25.3 | 4.01 | NR | NR | 43 |
| Salihi, 2019 | NR | NR | 34.6 | NR | NR | NR | NR | 42.3 |
| Shibata, 2015 | NR | NR | NR | NR | NR | 1.59 | 0.19 | NR |
| Smith, 2016 | NR | NR | NR | NR | NR | NR | NR | 51 |
| Solari, 2019 | NR | NR | 56.1 | NR | NR | NR | NR | NR |
| Tomšič, 2019 | NR | NR | 23.6 | NR | NR | NR | NR | 47.8 |
| Uchimuro, 2014 | NR | NR | 25.2 | NR | NR | NR | NR | NR |
| Vairo, 2023 | NR | NR | 63 | NR | NR | NR | NR | 62.5 |
| Van Leeuwen, 2013 | NR | NR | NR | NR | NR | NR | NR | NR |
| Van Praet, 2022 | NR | NR | 37.1 | 26 | 4.7 | 1.9 | 0.2 | NRNR |
| Wang, 2016 | NR | NR | NR | NR | NR | 1.81 | 0.2 | NR |
| Westhofen, 2016 | NR | NR | 43 | 25.1 | 3.4 | NR | NR | 44 |
| Zorinas, 2019 | 0.47 | 0.24-0.74 | 37.2 | 26.8 | 4.6 | NR | NR | 53.8 |
|  |  |  |  |  |  |  |  |  |
| Study/Year | **% Hyper-**  **cholesterolemia** | **% DM** | **% Smoker** | **% Prior Stroke** | **%Prior TIA** | **% Prior MI** | **% Afib** | **% Prior Cardiac Surgery** |
| Afshar, 2023 | 64 | 33.3 | 28 | NR | NR | NR | NR | NR |
| Agnino, 2022 | NR | 10.2 | NR | NR | NR | NR | 8.5 | NR |
| Agnino, 2019 | NR | NR | NR | NR | NR | NR | NR | 2.7 |
| Ascaso, 2023 | 27 | 6.7 | 23 | 5 | NR | NR | 29 | NR |
| Atluri, 2016 | NR | NR | NR | NR | NR | NR | NR | 9 |
| Balachandran, 2020 | NR | NR | NR | NR | NR | NR | 3.4 | NR |
| Bellitti, 2014 | NR | NR | NR | NR | NR | NR | 17.9 | NR |
| Berdajs, 2023 | 28 | 4.4 | 8.2 | 3.3 | NR | NR | 18 | NR |
| Chemtob, 2022 | 49.3 | 2.8 | 27.6 | 2.2 | NR | NR | 4.3 | NR |
| Chi, 2014 | NR | NR | NR | NR | NR | NR | NR | NR |
| D’Onofrio, 2022 | 16 | 3.5 | NR | NR | NR | NR | NR | NR |
| El-Andari, 2021 | 65.6 | 8.4 | 13.1 | NR | NR | NR | 8 | 2.9 |
| Franke, 2022 | NR | NR | NR | NR | NR | NR | NR | NR |
| Garatti, 2018 | NR | NR | NR | NR | NR | NR | NR | NR |
| Garcia Fuster, 2014 | 36.8 | 16.2 | 29.4 | NR | NR | NR | 32.4 | NR |
| Gerber, 2019 | NR | NR | NR | NR | NR | NR | NR | NR |
| Goldstone, 2015 | NR | NR | NR | NR | NR | NR | NR | 6.1 |
| Götte, 2022 | NR | 12.8 | 11.3 | 2.3 | NR | 4.3 | 42.8 | 5.5 |
| Grapsa, 2015 | NR | NR | NR | NR | NR | NR | 12.6 | NR |
| Grinberg, 2019 | NR | NR | NR | NR | NR | NR | NR | 0 |
| Güllü, 2021 | NR | NR | NR | NR | NR | NR | NR | NR |
| Hasham, 2022 | 39 | 3 | 38 | 2 | 2 | NR | 7 | NR |
| Hayashi, 2021 | NR | 8 | NR | NR | NR | NR | NR | NR |
| Hu, 2021 | NR | 4.8 | NR | NR | NR | NR | NR | NR |
| Huang, 2013 | NR | NR | NR | NR | NR | NR | NR | NR |
| Javadikasgari, 2017 | NR | 1.6 | NR | 1.6 | NR | NR | NR | NR |
| Kamiya, 2017 | NR | NR | NR | NR | NR | NR | 22.7 | NR |
| Kitai, 2014 | NR | NR | NR | NR | NR | NR | 29 | 0 |
| Kurlansky, 2023 | 18.9 | 12.4 | NR | 3.9 | 0.5 | 4.2 | NR | NR |
| Lange, 2017 | NR | NR | NR | NR | NR | 1.5 | NR | 2.4 |
| Lee, 2018 | NR | 10.5 | NR | NR | NR | NR | NR | NR |
| Levy, 2022 | 25 | NR | 37 | NR | NR | NR | 33 | NR |
| Li, 2022 | NR | 8.33 | NR | NR | NR | NR | 21.7 | 0.25 |
| Lio, 2017 | NR | NR | NR | NR | NR | NR | NR | NR |
| Ma, 2019 | NR | 4.6 | NR | 1.8 | NR | NR | NR | NR |
| Magruder, 2016 | NR | 4.5 | NR | 6.3 | NR | NR | 10.3 | 4.9 |
| Makarious, 2023 | 26.5 | 12.5 | 20.3 | 3.1 | NR | 3.1 | 46.9 | 1.6 |
| Mihaljevic, 2013 | NR | 1.2 | 34.7 | 1.2 | NR | NR | 5.1 | NR |
| Moscarelli, 2018 | 16.1 | NR | 3.2 | NR | NR | NR | NR | NR |
| Moscarelli, 2021 | NR | 20 | NR | NR | NR | NR | NR | NR |
| Muneretto, 2015 | 16 | NR | NR | 2 | NR | NR | 14 | 0 |
| Murzi, 2014 | 41.5 | 7.8 | 32.2 | NR | NR | 1.7 | 24.2 | NR |
| Noack, 2019 | NR | 19.7 | NR | NR | NR | NR | 38.6 | NR |
| Panos, 2015 | NR | NR | NR | NR | NR | NR | NR | 15 |
| Paranskaya, 2013 | NR | 7.7 | NR | 15.4 | NR | NR | 53.8 | 0 |
| Perin, 2019 | 22.2 | 4.63 | 7.41 | 2.78 | NR | 0 | NR | 0.9 |
| Petolat, 2023 | NR | 1 | NR | NR | NR | NR | 23 | NR |
| Ramzy, 2014 | NR | 4 | 24 | 3.7 | NR | NR | 33 | 0.3 |
| Salihi, 2019 | NR | 26.9 | NR | NR | NR | NR | 30.8 | 7.7 |
| Shibata, 2015 | NR | NR | NR | NR | NR | NR | 22 | 1.1 |
| Smith, 2016 | NR | 8 | NR | NR | NR | NR | 30 | NR |
| Solari, 2019 | NR | 21 | NR | NR | NR | NR | NR | 17.8 |
| Tomšič, 2019 | NR | 4.5 | NR | 5.4 | NR | NR | 36.2 | 3.35 |
| Uchimuro, 2014 | NR | NR | NR | NR | NR | NR | NR | NR |
| Vairo, 2023 | NR | 5.5 | NR | 2.7 | NR | NR | 32 | 8.5 |
| Van Leeuwen, 2013 | NR | NR | NR | NR | NR | NR | NR | NR |
| Van Praet, 2022 | NR | NR | NR | 2.9 | NR | NR | 33.6 | NR |
| Wang, 2016 | NR | NR | NR | NR | NR | NR | 14.4 | NR |
| Westhofen, 2016 | 6 | NR | NR | NR | NR | NR | 24 | 0 |
| Zorinas, 2019 | NR | 2.6 | NR | 3.8 | NR | NR | 6.4 | NR |
|  |  |  |  |  |  |  |  |  |
| Study/Year | **% Prior PAD** | **% Prior PVD** | **% Carotid Stenosis** | **% Aortic Stenosis** | **% Renal Failure** | **% CVD** | **% COPD** | **% Heart Failure** |
| Afshar, 2023 | NR | NR | NR | NR | NR | NR | NR | NR |
| Agnino, 2022 | NR | NR | NR | NR | NR | NR | 15.3 | NR |
| Agnino, 2019 | NR | NR | NR | NR | NR | NR | NR | NR |
| Ascaso, 2023 | NR | 2 | NR | NR | 5 | NR | 5 | NR |
| Atluri, 2016 | NR | NR | NR | NR | NR | NR | NR | NR |
| Balachandran, 2020 | NR | NR | NR | NR | NR | NR | NR | NR |
| Bellitti, 2014 | NR | NR | NR | NR | NR | NR | NR | NR |
| Berdajs, 2023 | NR | 1.1 | NR | NR | NR | NR | NR | NR |
| Chemtob, 2022 | 2 | NR | NR | NR | 0.1 | NR | 6.4 | NR |
| Chi, 2014 | NR | NR | NR | NR | NR | NR | NR | NR |
| D’Onofrio, 2022 | NR | NR | NR | NR | NR | NR | 4 | NR |
| El-Andari, 2021 | NR | 1.9 | NR | NR | 5 | NR | NR | 26.8 |
| Franke, 2022 | NR | NR | NR | NR | NR | NR | NR | NR |
| Garatti, 2018 | NR | NR | NR | NR | NR | NR | NR | NR |
| Garcia Fuster, 2014 | NR | NR | NR | NR | NR | 7.4 | 11.8 | NR |
| Gerber, 2019 | NR | NR | NR | NR | NR | NR | NR | NR |
| Goldstone, 2015 | 1.1 | NR | NR | NR | 1.3 | 5.3 | 8.4 | NR |
| Götte, 2022 | NR | NR | NR | NR | 0 | NR | 6.6 | NR |
| Grapsa, 2015 | NR | NR | NR | NR | NR | NR | NR | NR |
| Grinberg, 2019 | NR | NR | NR | NR | NR | NR | NR | NR |
| Güllü, 2021 | NR | NR | NR | NR | NR | NR | 6.7 | NR |
| Hasham, 2022 | NR | 1 | NR | NR | 0 | NR | 15 | NR |
| Hayashi, 2021 | NR | NR | NR | NR | 13 | NR | 5 | 30 |
| Hu, 2021 | NR | 1.9 | NR | NR | 1.6 | 5.6 | NR | NR |
| Huang, 2013 | NR | NR | NR | NR | NR | NR | NR | NR |
| Javadikasgari, 2017 | 1.1 | NR | NR | NR | NR | NR | 3.7 | NR |
| Kamiya, 2017 | NR | NR | NR | NR | NR | NR | NR | NR |
| Kitai, 2014 | NR | NR | NR | NR | NR | NR | NR | NR |
| Kurlansky, 2023 | 5.3 | NR | NR | NR | 1.8 | NR | NR | 44.9 |
| Lange, 2017 | NR | NR | NR | NR | NR | NR | NR | NR |
| Lee, 2018 | NR | NR | NR | NR | 9.9 | NR | NR | NR |
| Levy, 2022 | NR | NR | NR | NR | NR | NR | NR | 12 |
| Li, 2022 | NR | NR | NR | NR | NR | NR | NR | NR |
| Lio, 2017 | NR | NR | NR | NR | NR | NR | NR | NR |
| Ma, 2019 | NR | NR | NR | NR | 0.8 | NR | 1 | NR |
| Magruder, 2016 | NR | 0.5 | NR | NR | 0.9 | NR | NR | NR |
| Makarious, 2023 | NR | NR | NR | NR | NR | NR | 12.5 | NR |
| Mihaljevic, 2013 | 0.3 | NR | NR | NR | NR | 4.2 | 3.3 | 4.5 |
| Moscarelli, 2018 | NR | NR | NR | NR | NR | NR | NR | NR |
| Moscarelli, 2021 | NR | 7 | NR | NR | NR | NR | 11 | NR |
| Muneretto, 2015 | 8 | NR | NR | NR | 0 | NR | 2 | NR |
| Murzi, 2014 | NR | 4.2 | NR | NR | 2.7 | 3.5 | NR | NR |
| Noack, 2019 | 5.2 | NR | NR | NR | NR | NR | 5.2 | 100 |
| Panos, 2015 | NR | NR | NR | NR | NR | NR | NR | NR |
| Paranskaya, 2013 | NR | NR | NR | NR | 3.8 | NR | 7.7 | 50 |
| Perin, 2019 | NR | 3.7 | NR | NR | 4.63 | NR | 8.33 | NR |
| Petolat, 2023 | NR | NR | NR | NR | 1 | NR | 6 | MR |
| Ramzy, 2014 | NR | NR | NR | NR | NR | NR | NR | 36 |
| Salihi, 2019 | NR | 7.7 | NR | NR | NR | NR | 23.1 | NR |
| Shibata, 2015 | NR | NR | NR | NR | NR | NR | NR | NR |
| Smith, 2016 | NR | NR | NR | NR | 5 | NR | 11 | NR |
| Solari, 2019 | NR | NR | NR | NR | 8.9 | NR | NR | 22.3 |
| Tomšič, 2019 | 1.75 | NR | NR | NR | 11.8 | NR | 8.93 | NR |
| Uchimuro, 2014 | NR | NR | NR | NR | NR | NR | NR | NR |
| Vairo, 2023 | 8.5 | NR | NR | 0 | NR | NR | 7 | NR |
| Van Leeuwen, 2013 | NR | NR | NR | NR | NR | NR | NR | NR |
| Van Praet, 2022 | NR | NR | NR | NR | 13.4 | NR | NR | NR |
| Wang, 2016 | 0 | NR | NR | NR | NR | NR | NR | NR |
| Westhofen, 2016 | 5 | NR | NR | NR | 1 | NR | 5 | NR |
| Zorinas, 2019 | NR | NR | NR | NR | NR | NR | 3.8 | NR |
|  |  |  |  |  |  |  |  |  |
| Study/Year | **% Pacemaker** | **Mean LVEF** | **SD LVEF** | **% Moderate TR** | **% Severe TR** | **% Moderate AR** | **% Severe AR** | **% Mild MR** |
| Afshar, 2023 | NR | 37.8 | 8.2 | 28 | 2.7 | NR | NR | 37.3 |
| Agnino, 2022 | NR | 61.6 | 6.3 | NR | NR | NR | NR | NR |
| Agnino, 2019 | NR | 56.4 | 5.4 | NR | NR | NR | NR | NR |
| Ascaso, 2023 | NR | 61 | 7 | NR | NR | NR | NR | NR |
| Atluri, 2016 | NR | NR | NR | NR | NR | NR | NR | NR |
| Balachandran, 2020 | NR | 62 | NR | NR | NR | NR | NR | NR |
| Bellitti, 2014 | NR | NR | NR | NR | NR | NR | NR | NR |
| Berdajs, 2023 | NR | 60 | 8.8 | NR | NR | NR | NR | NR |
| Chemtob, 2022 | NR | NR | NR | NR | NR | 1.24 | 0.3 | NR |
| Chi, 2014 | NR | NR | NR | NR | NR | NR | NR | NR |
| D’Onofrio, 2022 | NR | 64 | 59.6-68.5 | NR | NR | NR | NR | 0 |
| El-Andari, 2021 | NR | NR | NR | NR | NR | NR | NR | NR |
| Franke, 2022 | NR | NR | NR | NR | NR | NR | NR | NR |
| Garatti, 2018 | NR | NR | NR | NR | NR | NR | NR | NR |
| Garcia Fuster, 2014 | NR | NR | NR | NR | NR | NR | NR | NR |
| Gerber, 2019 | NR | 68 | 60.5-73 | NR | NR | NR | NR | NR |
| Goldstone, 2015 | NR | 58.3 | 8.4 | 28.4 | NR | NR | NR | NR |
| Götte, 2022 | NR | 60 | 55-62 | NR | NR | NR | NR | NR |
| Grapsa, 2015 | NR | 58.07 | 10.6 | NR | NR | NR | NR | NR |
| Grinberg, 2019 | NR | 66 | 57-68 | NR | NR | NR | NR | NR |
| Güllü, 2021 | NR | NR | NR | NR | NR | NR | NR | NR |
| Hasham, 2022 | NR | 60.9 | 6.4 | NR | NR | NR | NR | NR |
| Hayashi, 2021 | NR | 68.5 | 9.2 | NR | NR | NR | NR | NR |
| Hu, 2021 | NR | 67.2 | 5.1 | NR | NR | NR | NR | NR |
| Huang, 2013 | NR | NR | NR | NR | NR | NR | NR | NR |
| Javadikasgari, 2017 | NR | NR | NR | NR | NR | NR | NR | NR |
| Kamiya, 2017 | NR | NR | NR | NR | NR | NR | NR | NR |
| Kitai, 2014 | NR | 64 | 10 | NR | NR | NR | NR | NR |
| Kurlansky, 2023 | NR | NR | NR | NR | NR | NR | NR | NR |
| Lange, 2017 | NR | 62.7 | 10.7 | NR | NR | NR | NR | NR |
| Lee, 2018 | NR | 70.2 | 6.8 | NR | NR | NR | NR | NR |
| Levy, 2022 | NR | 63 | 7 | NR | NR | NR | NR | NR |
| Li, 2022 | NR | 62.8 | 6.6 | NR | NR | NR | NR | NR |
| Lio, 2017 | NR | NR | NR | NR | NR | NR | NR | NR |
| Ma, 2019 | NR | 63.7 | 4.4 | NR | NR | NR | NR | NR |
| Magruder, 2016 | NR | 63 | 4 | NR | NR | NR | NR | NR |
| Makarious, 2023 | NR | NR | NR | NR | NR | NR | NR | NR |
| Mihaljevic, 2013 | NR | 59.7 | 4.3 | NR | 1.8 | NR | NR | NR |
| Moscarelli, 2018 | NR | NR | NR | NR | NR | NR | NR | NR |
| Moscarelli, 2021 | 7 | 32.3 | 6.4 | NR | NR | NR | NR | NR |
| Muneretto, 2015 | NR | 62 | 7 | NR | NR | NR | NR | NR |
| Murzi, 2014 | NR | 58.6 | 32 | NR | NR | NR | NR | NR |
| Noack, 2019 | NR | 36.8 | 9.4 | NR | NR | NR | NR | 0 |
| Panos, 2015 | NR | 60 | 8 | NR | NR | NR | NR | NR |
| Paranskaya, 2013 | NR | 58.8 | 8.2 | NR | NR | NR | NR | NR |
| Perin, 2019 | NR | NR | NR | NR | NR | NR | NR | NR |
| Petolat, 2023 | NR | 69 | 9 | NR | NR | NR | NR | NR |
| Ramzy, 2014 | NR | 61.1 | 8.3 | NR | NR | NR | NR | NR |
| Salihi, 2019 | NR | 59 | 3.1 | 3.8 | 0 | NR | NR | 0 |
| Shibata, 2015 | NR | 65 | 8 | NR | NR | NR | NR | NR |
| Smith, 2016 | NR | NR | NR | NR | NR | NR | NR | NR |
| Solari, 2019 | NR | NR | NR | NR | NR | NR | NR | NR |
| Tomšič, 2019 | NR | NR | NR | NR | NR | NR | NR | NR |
| Uchimuro, 2014 | NR | 65.9 | 7.1 | NR | NR | NR | NR | NR |
| Vairo, 2023 | NR | 63 | 5 | 18 | NR | 1.4 | NR | 0 |
| Van Leeuwen, 2013 | NR | NR | NR | NR | NR | NR | NR | NR |
| Van Praet, 2022 | NR | 59.3 | 5.9 | 7.3 | 0.6 | 0.6 | 0 | NR |
| Wang, 2016 | NR | 67.3 | 7 | NR | NR | NR | NR | NR |
| Westhofen, 2016 | NR | NR | NR | NR | NR | NR | NR | 0 |
| Zorinas, 2019 | 0 | 57 | 5 | 14.1 | NR | NR | NR | NR |
|  |  |  |  |  |  |  |  |  |
| Study/Year | **% Moderate MR** | **% Severe MR** | **Mean MR** | **SD MR** | **% MS** | **% Ischemic MR** | **% Degenerative MR** | **% MVP** |
| Afshar, 2023 | 62.7 | NR | NR | NR | 0 | 0 | 0 | 0 |
| Agnino, 2022 | NR | NR | NR | NR | NR | 0 | 100 | 100 |
| Agnino, 2019 | NR | NR | NR | NR | NR | 0 | 100 | 100 |
| Ascaso, 2023 | NR | NR | NR | NR | NR | 0 | 100 | NR |
| Atluri, 2016 | NR | NR | NR | NR | NR | NR | NR | NR |
| Balachandran, 2020 | NR | NR | NR | NR | NR | 0 | 100 | NR |
| Bellitti, 2014 | NR | NR | NR | NR | NR | 0 | 100 | 100 |
| Berdajs, 2023 | NR | NR | NR | NR | NR | 0.8 | 93.7 | 100 |
| Chemtob, 2022 | 6.2 | 93.8 | NR | NR | NR | 0 | 100 | NR |
| Chi, 2014 | NR | NR | NR | NR | NR | NR | NR | NR |
| D’Onofrio, 2022 | 0 | 100 | NR | NR | NR | 0 | 100 | NR |
| El-Andari, 2021 | NR | NR | NR | NR | NR | NR | NR | NR |
| Franke, 2022 | NR | NR | NR | NR | NR | NR | NR | NR |
| Garatti, 2018 | NR | NR | NR | NR | NR | 0 | 100 | NR |
| Garcia Fuster, 2014 | NR | 100 | NR | NR | NR | NR | 100 | 100 |
| Gerber, 2019 | NR | 100 | NR | NR | NR | NR | NR | NR |
| Goldstone, 2015 | NR | 96 | NR | NR | NR | 0 | 100 | 100 |
| Götte, 2022 | NR | NR | NR | NR | NR | NR | NR | NR |
| Grapsa, 2015 | NR | NR | NR | NR | NR | NR | NR | 100 |
| Grinberg, 2019 | NR | 100 | NR | NR | NR | NR | NR | 100 |
| Güllü, 2021 | NR | NR | NR | NR | NR | NR | NR | NR |
| Hasham, 2022 | 7 | 93 | NR | NR | NR | 0 | 100 | NR |
| Hayashi, 2021 | NR | NR | NR | NR | NR | NR | NR | NR |
| Hu, 2021 | NR | NR | NR | NR | NR | 0 | 100 | NR |
| Huang, 2013 | NR | NR | NR | NR | NR | 0 | 85.4 | NR |
| Javadikasgari, 2017 | NR | NR | NR | NR | NR | NR | NR | 100 |
| Kamiya, 2017 | NR | NR | NR | NR | NR | 0 | 100 | NR |
| Kitai, 2014 | NR | NR | NR | NR | NR | 0 | 100 | NR |
| Kurlansky, 2023 | NR | NR | NR | NR | NR | 0 | 100 | NR |
| Lange, 2017 | NR | NR | NR | NR | NR | NR | NR | NR |
| Lee, 2018 | NR | 78.9 | NR | NR | NR | 0 | 0 | NR |
| Levy, 2022 | NR | NR | NR | NR | NR | 0 | 100 | 100 |
| Li, 2022 | NR | NR | NR | NR | NR | NR | NR | 25 |
| Lio, 2017 | NR | NR | NR | NR | NR | NR | NR | NR |
| Ma, 2019 | NR | NR | NR | NR | NR | 0 | 100 | NR |
| Magruder, 2016 | NR | NR | NR | NR | NR | 0 | 100 | NR |
| Makarious, 2023 | NR | NR | NR | NR | NR | NR | NR | NR |
| Mihaljevic, 2013 | 0.5 | 96.4 | NR | NR | NR | 0 | 100 | NR |
| Moscarelli, 2018 | NR | NR | NR | NR | NR | 0 | 100 | NR |
| Moscarelli, 2021 | NR | 100 | NR | NR | NR | 17 | 0 | NR |
| Muneretto, 2015 | NR | 98 | NR | NR | NR | 0 | 100 | 100 |
| Murzi, 2014 | NR | NR | NR | NR | 0.3 | 9.2 | 86.4 | NR |
| Noack, 2019 | 4.1 | 95.9 | NR | NR | 0 | NR | NR | NR |
| Panos, 2015 | NR | NR | NR | NR | NR | 0 | 96.5 | NR |
| Paranskaya, 2013 | NR | NR | NR | NR | NR | NR | NR | 60 |
| Perin, 2019 | NR | NR | NR | NR | NR | 6.5 | 88.9 | 88.9 |
| Petolat, 2023 | NR | 100 | NR | NR | NR | 0 | 100 | 100 |
| Ramzy, 2014 | NR | 100 | NR | NR | NR | NR | NR | NR |
| Salihi, 2019 | 0 | 100 | NR | NR | 0 | 0 | 100 | 100 |
| Shibata, 2015 | 5 | 95 | NR | NR | NR | 0 | 89.4 | 100 |
| Smith, 2016 | NR | NR | NR | NR | NR | NR | NR | NR |
| Solari, 2019 | NR | NR | NR | NR | NR | 0 | 0 | NR |
| Tomšič, 2019 | NR | NR | NR | NR | NR | 0 | 100 | 100 |
| Uchimuro, 2014 | NR | 100 | NR | NR | 0 | 0 | 100 | 100 |
| Vairo, 2023 | 0 | 100 | NR | NR | 0 | 0 | 100 | 100 |
| Van Leeuwen, 2013 | NR | NR | NR | NR | NR | 0 | 4 | NR |
| Van Praet, 2022 | NR | NR | NR | NR | NR | NR | NR | NR |
| Wang, 2016 | 8.3 | 91.7 | 3.96 | 0.13 | 0 | 0 | 100 | 100 |
| Westhofen, 2016 | 0 | 100 | NR | NR | 0 | NR | NR | 92 |
| Zorinas, 2019 | NR | 100 | NR | NR | NR | 0 | 100 | NR |
|  |  |  |  |  |  |  |  |  |
| Study/Year | **% Anterior MVP** | **% Posterior MVP** | **% Bileaflet MVP** | **% Endocarditis** | **% Rheumatic** | **%Other Indication** |  |  |
| Afshar, 2023 | 0 | 0 | 0 | 100 | 0 | 0 |  |  |
| Agnino, 2022 | 3.4 | 71.2 | 27.1 | 0 | 0 | 0 |  |  |
| Agnino, 2019 | NR | NR | NR | 0 | 0 | 0 |  |  |
| Ascaso, 2023 | 8 | 64 | 28 | 0 | 0 | 0 |  |  |
| Atluri, 2016 | NR | NR | NR | NR | NR | NR |  |  |
| Balachandran, 2020 | NR | NR | NR | 0 | 0 | 0 |  |  |
| Bellitti, 2014 | 0 | 0 | 100 | 1.4 | 0 | 0 |  |  |
| Berdajs, 2023 | NR | NR | NR | 0 | 0 | 0 |  |  |
| Chemtob, 2022 | 6 | 69.7 | 23.6 | NR | NR | NR |  |  |
| Chi, 2014 | NR | NR | NR | 100 | NR | NR |  |  |
| D’Onofrio, 2022 | NR | NR | NR | NR | NR | NR |  |  |
| El-Andari, 2021 | NR | NR | NR | 0 | NR | NR |  |  |
| Franke, 2022 | NR | NR | NR | NR | NR | NR |  |  |
| Garatti, 2018 | NR | NR | NR | 0 | 0 | 0 |  |  |
| Garcia Fuster, 2014 | 41.2 | 80.9 | NR | 0 | 0 | 0 |  |  |
| Gerber, 2019 | NR | NR | NR | NR | NR | NR |  |  |
| Goldstone, 2015 | 5.5 | 75 | 19.4 | 0 | 0 | 0 |  |  |
| Götte, 2022 | NR | NR | NR | NR | NR | NR |  |  |
| Grapsa, 2015 | 0 | 100 | 0 | 0 | 0 | 0 |  |  |
| Grinberg, 2019 | NR | 100 | NR | NR | NR | NR |  |  |
| Güllü, 2021 | 13.3 | 70 | NR | NR | NR | NR |  |  |
| Hasham, 2022 | 7 | 66 | 27 | 0 | 0 | 0 |  |  |
| Hayashi, 2021 | NR | NR | NR | 8 | NR | NR |  |  |
| Hu, 2021 | NR | NR | NR | 0 | 0 | 0 |  |  |
| Huang, 2013 | 29.2 | 50 | 18.8 | 2.1 | 4.2 | 10.3 |  |  |
| Javadikasgari, 2017 | 0 | 100 | 0 | NR | NR | NR |  |  |
| Kamiya, 2017 | NR | NR | NR | 0 | 0 | 0 |  |  |
| Kitai, 2014 | 23.5 | 52.2 | 24.3 | 0 | 0 | 0 |  |  |
| Kurlansky, 2023 | 5 | 51 | 10 | 0 | 0 | 0 |  |  |
| Lange, 2017 | NR | NR | NR | NR | NR | NR |  |  |
| Lee, 2018 | 55.6 | 86.7 | 42.6 | 100 | 0 | 0 |  |  |
| Levy, 2022 | 0 | 0 | 100 | 0 | 0 | 0 |  |  |
| Li, 2022 | NR | NR | NR | NR | NR | NR |  |  |
| Lio, 2017 | 24 | 60 | 16 | NR | NR | NR |  |  |
| Ma, 2019 | 22.3 | 65.9 | 6.4 | 0 | 0 | 0 |  |  |
| Magruder, 2016 | 13 | 68.1 | 25 | 0 | 0 | 0 |  |  |
| Makarious, 2023 | NR | NR | NR | 0 | NR | NR |  |  |
| Mihaljevic, 2013 | NR | NR | NR | 0 | 0 | 0 |  |  |
| Moscarelli, 2018 | 14.5 | 61.3 | 22.5 | 0 | 0 | 0 |  |  |
| Moscarelli, 2021 | NR | NR | NR | NR | NR | NR |  |  |
| Muneretto, 2015 | 0 | 0 | 100 | 0 | 0 | 0 |  |  |
| Murzi, 2014 | NR | NR | NR | 3.2 | 1.2 | NR |  |  |
| Noack, 2019 | NR | NR | NR | 0 | 0 | NR |  |  |
| Panos, 2015 | 13.4 | 79 | 7.6 | 3.5 | 0 | 0 |  |  |
| Paranskaya, 2013 | 16 | 40 | 4 | 0 | NR | NR |  |  |
| Perin, 2019 | NR | NR | NR | 1.9 | 0 | 0 |  |  |
| Petolat, 2023 | NR | NR | NR | 0 | 0 | 0 |  |  |
| Ramzy, 2014 | 28.3 | NR | 19 | NR | NR | NR |  |  |
| Salihi, 2019 | NR | 84.6 | NR | 0 | 0 | NR |  |  |
| Shibata, 2015 | 26.7 | 47.7 | 25.6 | 0.6 | 3.3 | 6.7 |  |  |
| Smith, 2016 | NR | NR | NR | NR | NR | NR |  |  |
| Solari, 2019 | NR | NR | NR | 100 | 0 | 0 |  |  |
| Tomšič, 2019 | NR | NR | NR | 0 | 0 | 0 |  |  |
| Uchimuro, 2014 | 0 | 100 | 0 | 0 | 0 | 0 |  |  |
| Vairo, 2023 | NR | 91.6 | NR | 0 | 0 | 0 |  |  |
| Van Leeuwen, 2013 | 17 | 76 | NR | 11 | 0 | 0 |  |  |
| Van Praet, 2022 | NR | NR | NR | NR | NR | NR |  |  |
| Wang, 2016 | 13.1 | 78.6 | 8.33 | 0 | 0 | 0 |  |  |
| Westhofen, 2016 | 12 | 58 | 22 | 0 | 0 | 8 |  |  |
| Zorinas, 2019 | NR | NR | NR | 0 | 0 | 0 |  |  |

*Afib: Atrial Fibrillation; AR: Aortic Regurgitation; BMI: Body Mass Index; BSA: Body Surface Area; COPD: Chronic Obstructive Pulmonary Disease; CVD: Cerebrovascular Disease; DM: Diabetes Mellitus; LVEF: Left Ventricular Ejection Fraction; MI: Myocardial Infarction; MR: Mitral Regurgitation; MS: Mitral Stenosis; MVP: Mitral Valve Proplase; NR: Not Reported; PAD: Peripheral Arterial Disease; PVD: Peripheral Vascular Disease; SD: Standard Deviation; TIA: Transient Ischemic Attack; TR: Tricuspid Regurgitation*

**Supplemental Table 3.** Summary of procedure-related variables of the included studies.

| Study/Year | % Robotic | % Minimally Invasive | % Mini Sternotomy | % Mini Thoracotomy | % Other MI Approach | % Posterior Leaflet Repair | % Anterior Leaflet Repair | % Bileaflet Repair |
| --- | --- | --- | --- | --- | --- | --- | --- | --- |
| Afshar, 2023 | 0 | 0 | 0 | 0 | 0 | NR | NR | NR |
| Agnino, 2022 | 100 | 0 | 0 | 0 | 0 | NR | NR | NR |
| Agnino, 2019 | 0 | 100 | 0 | 100 | 0 | NR | NR | NR |
| Ascaso, 2023 | 0 | 63 | 0 | 63 | 0 | NR | NR | NR |
| Atluri, 2016 | 0 | 57.2 | 0 | 0 | 0 | NR | NR | NR |
| Balachandran, 2020 | NR | NR | NR | NR | NR | NR | NR | NR |
| Bellitti, 2014 | 0 | 0 | 0 | 0 | 0 | NR | NR | NR |
| Berdajs, 2023 | 0 | 100 | 0 | 0 | Endoscopic | 71.7 | 3.3 | 24.9 |
| Chemtob, 2022 | 60.5 | 0 | 0 | 0 | 0 | NR | NR | NR |
| Chi, 2014 | 100 | NR | NR | NR | NR | NR | NR | NR |
| D’Onofrio, 2022 | NR | 50 | NR | NR | Transapical Neochordae Implantation | NR | NR | NR |
| El-Andari, 2021 | NR | NR | NR | NR | NR | NR | NR | NR |
| Franke, 2022 | 100 | 0 | 0 | 0 | 0 | 60 | 5 | 32 |
| Garatti, 2018 | 0 | 0 | 0 | 0 | 0 | NR | NR | NR |
| Garcia Fuster, 2014 | 0 | 0 | 0 | 0 | 0 | NR | NR | NR |
| Gerber, 2019 | 0 | 100 | 0 | 0 | 0 | NR | NR | NR |
| Goldstone, 2015 | 0 | 46.3 | 0 | 0 | 0 | NR | NR | NR |
| Götte, 2022 | 0 | 61.1 | 0 | 0 | 0 | NR | NR | NR |
| Grapsa, 2015 | 0 | 0 | 0 | 0 | 0 | NR | NR | NR |
| Grinberg, 2019 | NR | 100 | NR | NR | Transapical Neochordae Implantation | NR | NR | NR |
| Güllü, 2021 | 100 | 0 | 0 | 0 | 0 | NR | NR | NR |
| Hasham, 2022 | 0 | 0 | 0 | 55 | NR | NR | NR | NR |
| Hayashi, 2021 | 0 | 100 | 0 | 0 | 0 | NR | NR | NR |
| Hu, 2021 | 0 | 33.5 | 0 | 0 | NR | NR | NR | NR |
| Huang, 2013 | NR | NR | NR | NR | NR | NR | NR | NR |
| Javadikasgari, 2017 | 98.7 | NR | NR | NR | NR | NR | NR | NR |
| Kamiya, 2017 | 0 | 95.5 | 0 | 0 | 0 | NR | NR | NR |
| Kitai, 2014 | NR | NR | NR | NR | NR | NR | NR | NR |
| Kurlansky, 2023 | 0 | 0 | 0 | 0 | 0 | NR | NR | NR |
| Lange, 2017 | NR | 100 | 32.8 | 67.2 | NR | NR | NR | NR |
| Lee, 2018 | 0 | 0 | 0 | 0 | 0 | NR | NR | NR |
| Levy, 2022 | 0 | 19 | 0 | 0 | NR | NR | NR | NR |
| Li, 2022 | 100 | 0 | 0 | 0 | 0 | NR | NR | NR |
| Lio, 2017 | 0 | 100 | 0 | 0 | Prosthetic Ring with Chordal Sizing System | NR | NR | NR |
| Ma, 2019 | NR | NR | 2.8 | 7.2 | NR | NR | NR | NR |
| Magruder, 2016 | 3.2 | 40.4 | 0 | 0 | 0 | NR | NR | NR |
| Makarious, 2023 | 0 | 0 | 0 | 0 | NR | NR | NR | NR |
| Mihaljevic, 2013 | 100 | 0 | 0 | 0 | 0 | NR | NR | NR |
| Moscarelli, 2018 | 0 | 100 | 0 | 0 | 0 | NR | NR | NR |
| Moscarelli, 2021 | 0 | 100 | 0 | 0 | 0 | NR | NR | NR |
| Muneretto, 2015 | 0 | 100 | 0 | 0 | 0 | NR | NR | NR |
| Murzi, 2014 | 0 | 100 | 0 | 0 | 0 | NR | NR | NR |
| Noack, 2019 | 0 | 58.7 | 0 | 58.7 | 0 | NR | NR | NR |
| Panos, 2015 | 0 | 100 | 0 | 0 | Minimally Invasive Video-Assisted Repair | NR | NR | NR |
| Paranskaya, 2013 | NR | NR | NR | NR | NR | NR | NR | NR |
| Perin, 2019 | 0 | 100 | 0 | 100 | 0 | NR | NR | NR |
| Petolat, 2023 | 0 | 0 | 0 | 0 | 0 | NR | NR | NR |
| Ramzy, 2014 | 99.7 | 0.3 | 0 | 0.3 | 0 | NR | NR | NR |
| Salihi, 2019 | 0 | 100 | 0 | 100 | Left Anterior Thoracotomy | NR | NR | NR |
| Shibata, 2015 | 0 | 0 | 0 | 0 | 0 | NR | NR | NR |
| Smith, 2016 | 0 | 41 | 11 | 30 | 0 | NR | NR | NR |
| Solari, 2019 | 0 | 0 | 0 | 0 | 0 | NR | NR | NR |
| Tomšič, 2019 | 0 | 0 | 0 | 0 | 0 | NR | NR | NR |
| Uchimuro, 2014 | 0 | 0 | 0 | 0 | 0 | 99.2 | 0 | 0 |
| Vairo, 2023 | 0 | 100 | 0 | 0 | 0 | NR | NR | NR |
| Van Leeuwen, 2013 | 0 | 0 | 0 | 0 | 0 | NR | NR | NR |
| Van Praet, 2022 | 0 | 100 | 0 | 100 | 0 | NR | NR | NR |
| Wang, 2016 | 100 | 0 | 0 | 0 | 0 | 78.6 | 31.1 | 8.33 |
| Westhofen, 2016 | 0 | 100 | 0 | 0 | 0 | NR | NR | NR |
| Zorinas, 2019 | 0 | 100 | 0 | 0 | 0 | NR | NR | NR |
| Study/Year | **% Quad Resection** | **% Edge to Edge** | **% Chord Implant** | **Mean number of chords** | **SD number of chords** | **% Chord transfer** | **% Wedge resection** | **% Ring** |
| Afshar, 2023 | 0 | 21.3 | 0 | 0 | NR | 20 | 0 | 100 |
| Agnino, 2022 | 0 | 10.2 | 10.2 | NR | NR | NR | NR | NR |
| Agnino, 2019 | NR | NR | NR | NR | NR | NR | NR | NR |
| Ascaso, 2023 | 12 | NR | 48.6 | NR | NR | NR | NR | NR |
| Atluri, 2016 | NR | NR | NR | NR | NR | NR | NR | NR |
| Balachandran, 2020 | NR | NR | NR | NR | NR | NR | NR | NR |
| Bellitti, 2014 | NR | NR | NR | NR | NR | NR | NR | NR |
| Berdajs, 2023 | NR | NR | NR | 1.7 | 0.91 | NR | NR | 17.4 |
| Chemtob, 2022 | NR | NR | 32.6 | NR | NR | NR | NR | NR |
| Chi, 2014 | NR | NR | NR | NR | NR | NR | NR | NR |
| D’Onofrio, 2022 | NR | NR | NR | NR | NR | NR | NR | NR |
| El-Andari, 2021 | NR | NR | 64.6 | NR | NR | NR | NR | 96.9 |
| Franke, 2022 | 27 | NR | 62 | NR | NR | NR | NR | 3 |
| Garatti, 2018 | NR | NR | NR | NR | NR | NR | NR | NR |
| Garcia Fuster, 2014 | NR | NR | NR | NR | NR | NR | NR | 100 |
| Gerber, 2019 | NR | NR | NR | NR | NR | NR | NR | 100 |
| Goldstone, 2015 | NR | 4.4 | 16 | NR | NR | NR | NR | NR |
| Götte, 2022 | NR | NR | 73.9 | NR | NR | NR | NR | NR |
| Grapsa, 2015 | NR | NR | NR | NR | NR | NR | NR | NR |
| Grinberg, 2019 | NR | NR | NR | 3 | 3-4 | NR | NR | NR |
| Güllü, 2021 | NR | 1.7 | NR | NR | NR | NR | NR | 11.7 |
| Hasham, 2022 | 27 | 0.3 | 60 | 1.88 | 0.83 | 0.07 | NR | 100 |
| Hayashi, 2021 | NR | 15 | 40 | NR | NR | NR | NR | NR |
| Hu, 2021 | NR | NR | 33.2 | NR | NR | NR | NR | 92.3 |
| Huang, 2013 | NR | NR | 100 | 1.9 | 0.7 | NR | NR | 100 |
| Javadikasgari, 2017 | 35 | NR | NR | NR | NR | NR | NR | NR |
| Kamiya, 2017 | 63.7 | NR | NR | NR | NR | NR | NR | NR |
| Kitai, 2014 | NR | NR | 48 | NR | NR | NR | NR | 89 |
| Kurlansky, 2023 | NR | NR | 1.8 | NR | NR | 28.8 | NR | 100 |
| Lange, 2017 | NR | NR | NR | NR | NR | NR | NR | NR |
| Lee, 2018 | 36.8 | NR | 5.3 | NR | NR | 36.8 | NR | 89.5 |
| Levy, 2022 | NR | NR | NR | NR | NR | NR | NR | 100 |
| Li, 2022 | NR | NR | NR | NR | NR | NR | NR | 100 |
| Lio, 2017 | NR | NR | NR | NR | NR | NR | NR | NR |
| Ma, 2019 | 12.8 | 11.3 | 38.2 | NR | NR | NR | NR | 65.9 |
| Magruder, 2016 | NR | NR | NR | NR | NR | NR | NR | NR |
| Makarious, 2023 | NR | NR | NR | NR | NR | NR | NR | NR |
| Mihaljevic, 2013 | NR | 1.2 | 25.7 | NR | NR | NR | NR | NR |
| Moscarelli, 2018 | NR | NR | 62.9 | NR | NR | NR | NR | 100 |
| Moscarelli, 2021 | NR | NR | NR | NR | NR | NR | NR | 100 |
| Muneretto, 2015 | NR | NR | 62 | NR | NR | NR | NR | 100 |
| Murzi, 2014 | NR | NR | NR | NR | NR | NR | NR | NR |
| Noack, 2019 | NR | NR | NR | NR | NR | NR | NR | 95 |
| Panos, 2015 | NR | NR | 100 | NR | NR | NR | NR | NR |
| Paranskaya, 2013 | NR | NR | NR | NR | NR | NR | NR | NR |
| Perin, 2019 | NR | NR | NR | NR | NR | NR | NR | 100 |
| Petolat, 2023 | NR | NR | 76 | NR | NR | NR | NR | 100 |
| Ramzy, 2014 | 17.7 | 14.7 | 11 | NR | NR | 16.7 | 62.7 | 100 |
| Salihi, 2019 | NR | NR | 100 | 3.12 | 0.8 | NR | NR | 0 |
| Shibata, 2015 | NR | 43.3 | NR | NR | NR | NR | NR | NR |
| Smith, 2016 | NR | NR | 100 | NR | NR | NR | NR | NR |
| Solari, 2019 | NR | NR | NR | NR | NR | NR | NR | 40.6 |
| Tomšič, 2019 | NR | NR | NR | NR | NR | NR | NR | NR |
| Uchimuro, 2014 | NR | NR | 21.8 | NR | NR | NR | NR | 100 |
| Vairo, 2023 | NR | NR | NR | NR | NR | NR | NR | NR |
| Van Leeuwen, 2013 | 78 | NR | 9 | NR | NR | 11 | NR | 98 |
| Van Praet, 2022 | NR | NR | 100 | 3.56 | NR | NR | NR | NR |
| Wang, 2016 | NR | 0 | 23.8 | NR | NR | NR | NR | 39.3 |
| Westhofen, 2016 | NR | 8 | 56 | NR | NR | NR | NR | 98 |
| Zorinas, 2019 | NR | NR | 100 | 3.5 | NR | NR | NR | NR |
|  |  |  |  |  |  |  |  |  |
| Study/Year | **% Band** | **% Leaflet Resection** | **% Leaflet Preserve** | **% Other Repair** | **Mean Annulus size** | **SD Annulus size** | **% MAC** | **Mean Operative time (min)** |
| Afshar, 2023 | 0 | 0 | 0 | 0 | NR | NR | NR | NR |
| Agnino, 2022 | NR | 5.1 | 66.1 | NR | NR | NR | NR | NR |
| Agnino, 2019 | NR | NR | NR | Free Margin Running Suture | NR | NR | NR | NR |
| Ascaso, 2023 | NR | NR | NR | NR | NR | NR | NR | NR |
| Atluri, 2016 | NR | NR | NR | NR | NR | NR | NR | NR |
| Balachandran, 2020 | NR | NR | NR | NR | NR | NR | NR | NR |
| Bellitti, 2014 | NR | NR | NR | NR | NR | NR | NR | NR |
| Berdajs, 2023 | NR | 0 | NR | NR | NR | NR | NR | NR |
| Chemtob, 2022 | NR | 63.8 | NR | NR | NR | NR | NR | NR |
| Chi, 2014 | NR | NR | NR | NR | NR | NR | NR | NR |
| D’Onofrio, 2022 | NR | NR | NR | NR | NR | NR | NR | 180 |
| El-Andari, 2021 | NR | 42.5 | NR | NR | NR | NR | NR | NR |
| Franke, 2022 | NR | 55 | 45 | NR | NR | NR | NR | NR |
| Garatti, 2018 | NR | NR | NR | NR | NR | NR | NR | NR |
| Garcia Fuster, 2014 | NR | NR | NR | NR | NR | NR | NR | NR |
| Gerber, 2019 | NR | NR | NR | NR | NR | NR | NR | NR |
| Goldstone, 2015 | NR | 68 | NR | NR | NR | NR | NR | NR |
| Götte, 2022 | NR | NR | NR | NR | NR | NR | NR | 183 |
| Grapsa, 2015 | NR | NR | NR | NR | NR | NR | NR | NR |
| Grinberg, 2019 | NR | NR | NR | NR | NR | NR | NR | 117 |
| Güllü, 2021 | NR | 38.3 | NR | NR | NR | NR | NR | NR |
| Hasham, 2022 | NR | 43.2 | NR | NR | 34 | 2.3 | NR | 138.3 |
| Hayashi, 2021 | NR | 29 | NR | Folding | NR | NR | NR | 211 |
| Hu, 2021 | NR | 83.5 | NR | NR | NR | NR | NR | NR |
| Huang, 2013 | NR | NR | NR | NR | NR | NR | NR | NR |
| Javadikasgari, 2017 | NR | 79 | NR | NR | NR | NR | NR | NR |
| Kamiya, 2017 | NR | NR | NR | NR | NR | NR | NR | 224.7 |
| Kitai, 2014 | NR | 86 | NR | NR | NR | NR | NR | NR |
| Kurlansky, 2023 | NR | 60.7 | NR | NR | NR | NR | NR | NR |
| Lange, 2017 | NR | NR | NR | NR | NR | NR | NR | NR |
| Lee, 2018 | NR | 81.6 | NR | NR | NR | NR | NR | NR |
| Levy, 2022 | NR | NR | NR | NR | NR | NR | NR | NR |
| Li, 2022 | NR | NR | NR | NR | NR | NR | NR | 320.4 |
| Lio, 2017 | NR | NR | NR | NR | NR | NR | NR | NR |
| Ma, 2019 | 34.1 | 37.2 | NR | NR | 29.1 | 2.1 | NR | NR |
| Magruder, 2016 | NR | NR | NR | NR | NR | NR | NR | NR |
| Makarious, 2023 | NR | NR | NR | NR | NR | NR | NR | 166.5 |
| Mihaljevic, 2013 | NR | 74.3 | NR | NR | NR | NR | NR | NR |
| Moscarelli, 2018 | NR | 48.3 | NR | NR | NR | NR | NR | 321.1 |
| Moscarelli, 2021 | NR | NR | NR | NR | NR | NR | NR | NR |
| Muneretto, 2015 | NR | NR | 38 | NR | NR | NR | NR | 258 |
| Murzi, 2014 | NR | NR | NR | NR | NR | NR | NR | NR |
| Noack, 2019 | 5 | NR | NR | NR | NR | NR | NR | NR |
| Panos, 2015 | NR | 9.4 | NR | NR | NR | NR | NR | NR |
| Paranskaya, 2013 | NR | NR | NR | NR | NR | NR | NR | NR |
| Perin, 2019 | NR | NR | NR | Resection and Chords | 32.8 | 2.7 | NR | NR |
| Petolat, 2023 | NR | 24 | NR | NR | NR | NR | NR | NR |
| Ramzy, 2014 | NR | NR | NR | Cleft Closure | 34.7 | 3 | NR | NR |
| Salihi, 2019 | 0 | NR | NR | Neochord | NR | NR | NR | 133 |
| Shibata, 2015 | NR | 1.7 | NR | Loop Technique | NR | NR | NR | NR |
| Smith, 2016 | 100 | NR | NR | NR | NR | NR | NR | NR |
| Solari, 2019 | 10.3 | 41.9 | NR | NR | NR | NR | NR | NR |
| Tomšič, 2019 | NR | NR | NR | Annulus Decalcification | NR | NR | 12 | NR |
| Uchimuro, 2014 | NR | 99.2 | NR | NR | NR | NR | NR | NR |
| Vairo, 2023 | NR | NR | NR | NR | 33.9 | 5.2 | 9.7 | NR |
| Van Leeuwen, 2013 | NR | NR | NR | NR | NR | NR | 4 | NR |
| Van Praet, 2022 | NR | NR | NR | NR | NR | NR | NR | NR |
| Wang, 2016 | 54.8 | 79.8 | NR | NR | 30.5 | NR | NR | NR |
| Westhofen, 2016 | NR | 58 | NR | Sliding Plasty | NR | NR | NR | 257.7 |
| Zorinas, 2019 | NR | NR | NR | NR | NR | NR | NR | 120 |
| Study/Year | **SD Operative Time (min)** | **% Cross clamp** | **Mean cross clamp time** | **Sd cross clamp time** | **% Bypass** | **Mean Bypass time** | **SD bypass time** | **% convert to replacement** |
| Afshar, 2023 | NR | NR | NR | NR | NR | NR | NR | 0 |
| Agnino, 2022 | NR | NR | 100.5 | 27.3 | NR | 177.3 | 39.5 | NR |
| Agnino, 2019 | NR | NR | 108 | 31 | NR | 149 | 37 | NR |
| Ascaso, 2023 | NR | NR | 88 | 64-105 | NR | 115 | 85-134 | 1.7 |
| Atluri, 2016 | NR | NR | NR | NR | NR | NR | NR | NR |
| Balachandran, 2020 | NR | NR | NR | NR | NR | NR | NR | NR |
| Bellitti, 2014 | NR | NR | NR | NR | NR | NR | NR | NR |
| Berdajs, 2023 | NR | NR | 95 | 27 | NR | 153 | 52 | 0 |
| Chemtob, 2022 | NR | NR | 65.9 | 19.6 | NR | 103.6 | 29.8 | 0.2 |
| Chi, 2014 | NR | NR | 89 | NR | NR | 124 | NR | NR |
| D’Onofrio, 2022 | 120-240 | NR | NR | NR | NR | NR | NR | 0 |
| El-Andari, 2021 | NR | NR | NR | 113.6 | 34.7 | NR | NR | NR |
| Franke, 2022 | NR | NR | 88 | 28 | NR | 166 | 50 | NR |
| Garatti, 2018 | NR | NR | NR | NR | NR | NR | NR | NR |
| Garcia Fuster, 2014 | NR | NR | NR | NR | NR | NR | NR | NR |
| Gerber, 2019 | NR | NR | NR | NR | NR | NR | NR | 2.6 |
| Goldstone, 2015 | NR | NR | 93.6 | 36.3 | NR | 96.1 | 47.2 | 1 |
| Götte, 2022 | 159-218 | NR | 70 | 57-86 | NR | 121 | 90-148 | NR |
| Grapsa, 2015 | NR | NR | NR | NR | NR | NR | NR | NR |
| Grinberg, 2019 | 114-126 | NR | NR | NR | NR | NR | NR | NR |
| Güllü, 2021 | NR | NR | NR | NR | NR | NR | NR | NR |
| Hasham, 2022 | 30.1 | NR | 65.3 | 14.2 | NR | 89.6 | 20.8 | NR |
| Hayashi, 2021 | 42 | NR | 108 | 31 | NR | 134 | 35 | NR |
| Hu, 2021 | NR | NR | NR | NR | NR | NR | NR | NR |
| Huang, 2013 | NR | NR | 77.3 | 18.5 | NR | 119.4 | 23.4 | NR |
| Javadikasgari, 2017 | NR | NR | NR | NR | NR | NR | NR | NR |
| Kamiya, 2017 | 51.7 | NR | 114.6 | 32 | NR | 172.4 | 47.8 | NR |
| Kitai, 2014 | NR | NR | NR | NR | NR | NR | NR | NR |
| Kurlansky, 2023 | NR | NR | NR | NR | NR | NR | NR | NR |
| Lange, 2017 | NR | NR | 81.4 | 26.3 | NR | 113.1 | 31.7 | NR |
| Lee, 2018 | NR | 100 | 98.9 | 41.4 | 100 | 136.1 | 50.9 | NR |
| Levy, 2022 | NR | 100 | 49 | 19 | 100 | 74 | 27 | NR |
| Li, 2022 | 78.6 | 100 | 95.13 | 45.64 | 100 | 152.32 | 45.77 | 0 |
| Lio, 2017 | NR | NR | 102 | 59 | NR | 167 | 79 | NR |
| Ma, 2019 | NR | 100 | 80.6 | 24.7 | 100 | 119.7 | 35.9 | NR |
| Magruder, 2016 | NR | NR | NR | NR | NR | 115 | 40 | NR |
| Makarious, 2023 | 107-275 | 100 | 58 | 33-122 | 100 | 90.5 | 56-175 | NR |
| Mihaljevic, 2013 | NR | NR | NR | NR | NR | 119 | 34 | NR |
| Moscarelli, 2018 | 45.5 | 100 | 92.6 | 29.5 | 100 | 140 | 39 | NR |
| Moscarelli, 2021 | NR | 100 | 31 | 9.6 | 100 | 61 | 17.2 | NR |
| Muneretto, 2015 | 48 | 98 | 23 | NR | NR | 131 | 41 | NR |
| Murzi, 2014 | NR | NR | 88.6 | 40.4 | NR | 128.6 | 53.6 | 11.8 |
| Noack, 2019 | NR | 100 | 62.9 | 27.3 | 100 | 111.7 | 39.4 | 0 |
| Panos, 2015 | NR | NR | 106 | 28 | NR | 133 | 30 | 0.1 |
| Paranskaya, 2013 | NR | NR | NR | NR | NR | NR | NR | NR |
| Perin, 2019 | NR | 100 | 122.9 | 44.6 | 100 | 183.1 | 33.7 | 0 |
| Petolat, 2023 | NR | NR | 55 | 18 | NR | NR | NR | NR |
| Ramzy, 2014 | NR | 100 | 101 | 25.2 | 100 | 144 | 35.2 | 0 |
| Salihi, 2019 | 15.2 | 0 | NR | NR | 0 | NR | NR | 3.8 |
| Shibata, 2015 | NR | NR | NR | NR | NR | NR | NR | NR |
| Smith, 2016 | NR | NR | 87 | 23 | NR | 100 | 28 | NR |
| Solari, 2019 | NR | NR | 90 | 31.3 | NR | 128 | 51.2 | NR |
| Tomšič, 2019 | NR | NR | NR | NR | NR | NR | NR | 1.44 |
| Uchimuro, 2014 | NR | NR | NR | NR | NR | NR | NR | NR |
| Vairo, 2023 | NR | NR | NR | NR | NR | NR | NR | NR |
| Van Leeuwen, 2013 | NR | NR | NR | NR | NR | NR | NR | 0 |
| Van Praet, 2022 | NR | NR | NR | NR | NR | NR | NR | NR |
| Wang, 2016 | NR | NR | NR | NR | NR | NR | NR | 0 |
| Westhofen, 2016 | 53.85 | 100 | 120 | 40 | 100 | 181.9 | 47.6 | 0 |
| Zorinas, 2019 | 110-146 | 0 | NR | NR | 0 | NR | NR | 0 |
|  |  |  |  |  |  |  |  |  |
|  |  |  |  |  |  |  |  |  |
| Study/Year | **Mean post-op LVEF** | **SD post-op LVEF** | **Mean hospital LOS** | **SD hospital LOS** |  |  |  |  |
| Afshar, 2023 | 36.2 | 8.5 | NR | NR |  |  |  |  |
| Agnino, 2022 | 57.3 | 8.7 | 6.8 | 3.4 |  |  |  |  |
| Agnino, 2019 | 54.6 | 5.2 | NR | NR |  |  |  |  |
| Ascaso, 2023 | NR | NR | 9 | 6-10 |  |  |  |  |
| Atluri, 2016 | NR | NR | 6.4 | NR |  |  |  |  |
| Balachandran, 2020 | NR | NR | NR | NR |  |  |  |  |
| Bellitti, 2014 | NR | NR | NR | NR |  |  |  |  |
| Berdajs, 2023 | 55 | 7.6 | 8 | 7-10 |  |  |  |  |
| Chemtob, 2022 | NR | NR | 5.5 | 2.7 |  |  |  |  |
| Chi, 2014 | NR | NR | NR | NR |  |  |  |  |
| D’Onofrio, 2022 | 59.5 | 55.3-66.3 | 7 | 7-9 |  |  |  |  |
| El-Andari, 2021 | NR | NR | NR | NR |  |  |  |  |
| Franke, 2022 | NR | NR | 7.6 | 5.3 |  |  |  |  |
| Garatti, 2018 | NR | NR | NR | NR |  |  |  |  |
| Garcia Fuster, 2014 | NR | NR | NR | NR |  |  |  |  |
| Gerber, 2019 | 60 | 55-66 | NR | NR |  |  |  |  |
| Goldstone, 2015 | 57.6 | 11.3 | NR | NR |  |  |  |  |
| Götte, 2022 | NR | NR | 13 | 12-16 |  |  |  |  |
| Grapsa, 2015 | 57.2 | 10.2 | NR | NR |  |  |  |  |
| Grinberg, 2019 | NR | NR | 9 | 8-13 |  |  |  |  |
| Güllü, 2021 | NR | NR | NR | NR |  |  |  |  |
| Hasham, 2022 | NR | NR | 3.4 | NR |  |  |  |  |
| Hayashi, 2021 | NR | NR | 11.3 | 10.7 |  |  |  |  |
| Hu, 2021 | NR | NR | NR | NR |  |  |  |  |
| Huang, 2013 | NR | NR | NR | NR |  |  |  |  |
| Javadikasgari, 2017 | NR | NR | 4.9 | 1.6 |  |  |  |  |
| Kamiya, 2017 | NR | NR | NR | NR |  |  |  |  |
| Kitai, 2014 | NR | NR | NR | NR |  |  |  |  |
| Kurlansky, 2023 | NR | NR | NR | NR |  |  |  |  |
| Lange, 2017 | NR | NR | 8.4 | 5.3 |  |  |  |  |
| Lee, 2018 | NR | NR | NR | NR |  |  |  |  |
| Levy, 2022 | NR | NR | 11 | 3 |  |  |  |  |
| Li, 2022 | 59.5 | 7 | NR | NR |  |  |  |  |
| Lio, 2017 | NR | NR | NR | NR |  |  |  |  |
| Ma, 2019 | NR | NR | NR | NR |  |  |  |  |
| Magruder, 2016 | NR | NR | 8 | 7 |  |  |  |  |
| Makarious, 2023 | NR | NR | NR | NR |  |  |  |  |
| Mihaljevic, 2013 | NR | NR | NR | NR |  |  |  |  |
| Moscarelli, 2018 | NR | NR | 8 | 2 |  |  |  |  |
| Moscarelli, 2021 | NR | NR | 8 | 6 |  |  |  |  |
| Muneretto, 2015 | NR | NR | 4.9 | 3.3 |  |  |  |  |
| Murzi, 2014 | NR | NR | NR | NR |  |  |  |  |
| Noack, 2019 | NR | NR | NR | NR |  |  |  |  |
| Panos, 2015 | NR | NR | 5 | 1.3 |  |  |  |  |
| Paranskaya, 2013 | 54.6 | 5.3 | 10.6 | 1.7 |  |  |  |  |
| Perin, 2019 | NR | NR | 6 | 1.85 |  |  |  |  |
| Petolat, 2023 | 55 | 9 | NR | NR |  |  |  |  |
| Ramzy, 2014 | NR | NR | 6 | 2.9 |  |  |  |  |
| Salihi, 2019 | NR | NR | 6.11 | 2.12 |  |  |  |  |
| Shibata, 2015 | NR | NR | NR | NR |  |  |  |  |
| Smith, 2016 | NR | NR | NR | NR |  |  |  |  |
| Solari, 2019 | NR | NR | NR | NR |  |  |  |  |
| Tomšič, 2019 | NR | NR | NR | NR |  |  |  |  |
| Uchimuro, 2014 | 54.1 | 9.06 | NR | NR |  |  |  |  |
| Vairo, 2023 | NR | NR | NR | NR |  |  |  |  |
| Van Leeuwen, 2013 | NR | NR | 9.7 | 5.7 |  |  |  |  |
| Van Praet, 2022 | NR | NR | NR | NR |  |  |  |  |
| Wang, 2016 | NR | NR | NR | NR |  |  |  |  |
| Westhofen, 2016 | NR | NR | 9 | 2.3 |  |  |  |  |
| Zorinas, 2019 | NR | NR | 8 | 7-9 |  |  |  |  |

*LVEF: Left Ventricular Ejection Fraction; LOS: Length of Stay; MAC: Mitral Annular Calcification; MI: Minimally Invasive; SD: Standard Deviation*

**Supplemental Table 4.** Summary of critical appraisal of included observational studies using the Newcastle Ottawa Quality Assessment Scale for Cohort Studies.

| Study/year | Selection | Comparability | Outcome |
| --- | --- | --- | --- |
| *Afshar/2023* | **** | - | *** |
| *Agnino/2022* | **** | - | *** |
| *Agnino/2019* | **** | - | *** |
| *Ascaso/2023* | **** | ** | *** |
| *Atluri/2016* | **** | ** | *** |
| *Balachandran/2020* | **** | - | *** |
| *Bellitti/2014* | **** | - | *** |
| *Berdajs/2023* | **** | - | *** |
| *Chemtob/2022* | **** | * | *** |
| *Chi/2014* | **** | - | *** |
| *D'Onofrio/2022* | **** | ** | *** |
| *ElAndari/2021* | **** | - | *** |
| *Franke/2022* | **** | * | *** |
| *Fuster/2014* | **** | - | *** |
| *Garatti/2018* | **** | ** | *** |
| *Gerber/2019* | **** | - | *** |
| *Goldstone/2015* | **** | ** | *** |
| *Götte/2022* | **** | ** | *** |
| *Grapsa/2015* | **** | - | *** |
| *Grinberg/2019* | **** | - | *** |
| *Güllü/2021* | **** | - | *** |
| *Hashim/2022* | **** | ** | *** |
| *Hayashi/2021* | **** | - | *** |
| *Hu/2021* | **** | ** | *** |
| *Huang/2013* | **** | ** | *** |
| *Javadikasgari/2017* | **** | - | *** |
| *Kamiya/2017* | **** | - | *** |
| *Kitai/2014* | **** | - | *** |
| *Kurlansky/2023* | **** | ** | *** |
| *Laham/2023* | **** | ** | *** |
| *Lange/2017* | **** | ** | *** |
| *Lee/2018* | **** | ** | *** |
| *Levy/2022* | **** | ** | *** |
| *Li/2022* | **** | - | *** |
| *Lio/2017* | **** | - | *** |
| *Ma/2019* | **** | - | *** |
| *Magruder/2016* | **** | ** | *** |
| *Mihaljevic/2013* | **** | ** | *** |
| *Moscarelli/2021* | **** | - | *** |
| *Muneretto/2015* | **** | - | *** |
| *Murzi/2014* | **** | * | *** |
| *Noack/2019* | **** | ** | *** |
| *Panos/2015* | **** | - | *** |
| *Paranskaya/2013* | **** | * | *** |
| *Perin/2019* | **** | ** | *** |
| *Petolat/2023* | **** | - | *** |
| *Ramzy/2014* | **** | ** | *** |
| *Salihi/2019* | **** | - | *** |
| *Shibata/2015* | **** | - | *** |
| *Smith/2016* | **** | - | *** |
| *Solari/2019* | **** | ** | *** |
| *Tomšič/2019* | **** | * | *** |
| *Uchimuro/2014* | **** | ** | *** |
| *Vairo/2023* | **** | - | *** |
| *van Leeuwen/2013* | **** | - | *** |
| *Van Praet/2022* | **** | - | *** |
| *Wang/2016* | **** | - | *** |
| *Westhofen/2016* | **** | ** | *** |
| *Zorinas/2019* | **** | * | *** |

**Selection**

 1)        Representativeness of intervention cohort a) truly representative of the average in the community *; b) somewhat representative of the average; c) only selected group of users; d) no description of the derivation of the cohort.

2)        Selection of nonintervention cohort – a) drawn from same community as intervention cohort*; b) drawn from a different source; c) no description of the derivation of the nonexposed cohort.

3)        Ascertainment of exposure a) secure record*; b) structured interview*; c) written selfreport; d) no description.

4)        Demonstration that outcome of interest was not present at start of study a) yes*; b) no.

**Comparability**

 1)       Comparability of cohorts on the basis of the design or analysis a) study controls for age, and gender*; b) study controls for any additional factor***.**

**Outcome**

 1)       Assessment of outcome a) independent blind assessment***;**b) record linkage*; c) selfreport; d) no description.

2)       Was followup long enough for outcomes to occur a) yes*; b) no

3)       Adequacy of follow up of cohorts a) complete follow up***;**b) subjects lost to follow up unlikely to introduce bias < 20 % lost follow up***;**c) follow up rate < 80% and no description of those lost; d) no statement.

| **Supplemental Table 5.** Summary of critical appraisal of included randomized control trials using version 2 of the Cochrane risk ofbias tool for randomized trials.  **Study details**   \| **Reference** \| Moscarelli M, Terrasini N, Nunziata A, Punjabi P, Angelini G, Solinas M, et al. A Trial of Two Anesthetic Regimes for Minimally Invasive Mitral Valve Repair. Journal of Cardiothoracic and Vascular Anesthesia. 2018 Dec;32(6):2562–9.  **MINI-SEVO Trial** \| \| --- \| --- \|   **Study design**   \| X \| Individually randomized parallel group trial \| \| --- \| --- \| \| □ \| Cluster randomized parallel group trial \| \| □ \| Individually randomized crossover (or other matched) trial \|   **For the purposes of this assessment, the interventions being compared are defined as**   \| Experimental: \| Sevoflurane Anesthetic \| Comparator: \| Propofol Anesthetic \| \| --- \| --- \| --- \| --- \|  \| **Specify which outcome is being assessed for risk of bias** \| Cardioprotection as measured by troponin release \| \| --- \| --- \|  \| **Specify the numerical result being assessed.** In case of multiple alternative analyses being presented, specify the numeric result (e.g. RR = 1.52 (95% CI 0.83 to 2.77) and/or a reference (e.g. to a table, figure or paragraph) that uniquely defines the result being assessed. \| Manuscript results in Figure 2. \| \| --- \| --- \|   **Is the review team’s aim for this result…?**   \| □ \| to assess the effect of *assignment to intervention* (the ‘intentiontotreat’ effect) \| \| --- \| --- \| \| X \| to assess the effect of *adhering to intervention* (the ‘perprotocol’ effect) \|   **If the aim is to assess the effect of *adhering to intervention***, select the deviations from intended intervention that should be addressed (at least one must be checked):  X occurrence of nonprotocol interventions  X failures in implementing the intervention that could have affected the outcome  □ nonadherence to their assigned intervention by trial participants  **Which of the following sources were obtained to help inform the risk of bias assessment? (tick as many as apply)**  X Journal article(s) with results of the trial  □ Trial protocol  □ Statistical analysis plan (SAP)  □ Noncommercial trial registry record (e.g. ClinicalTrials.gov record)  □ Companyowned trial registry record (e.g. GSK Clinical Study Register record)  □ “Grey literature” (e.g. unpublished thesis)  □ Conference abstract(s) about the trial  □ Regulatory document (e.g. Clinical Study Report, Drug Approval Package)  □ Research ethics application  □ Grant database summary (e.g. NIH RePORTER or Research Councils UK Gateway to Research)  □ Personal communication with trialist  □ Personal communication with the sponsor |
| --- | --- | --- | --- | --- | --- | --- | --- | --- | --- | --- | --- | --- | --- | --- | --- | --- | --- | --- | --- | --- |

Risk of bias assessment

Responses underlined in green are potential markers for low risk of bias, and responses in red are potential markers for a risk of bias. Where questions relate only to sign posts to other questions, no formatting is used.

**Domain 1: Risk of bias arising from the randomization process**

| **Signalling questions** | **Comments** | **Response options** |
| --- | --- | --- |
| **1.1 Was the allocation sequence random?** |  | Y |
| **1.2 Was the allocation sequence concealed until participants were enrolled and assigned to interventions?** |  | Y |
| **1.3 Did baseline differences between intervention groups suggest a problem with the randomization process?** |  | N |
| **Riskofbias judgement** |  | Low |
| Optional: What is the predicted direction of bias arising from the randomization process? |  | Unpredictable |

Domain 2: Risk of bias due to deviations from the intended interventions (*effect of assignment to intervention*)

| **Signalling questions** | **Comments** | **Response options** |
| --- | --- | --- |
| **2.1. Were participants aware of their assigned intervention during the trial?** |  | N |
| **2.2. Were carers and people delivering the interventions aware of participants' assigned intervention during the trial?** |  | Y |
| **2.3. If Y/PY/NI to 2.1 or 2.2: Were there deviations from the intended intervention that arose because of the trial context?** |  | N |
| **2.4 If Y/PY to 2.3: Were these deviations likely to have affected the outcome?** |  |  |
| **2.5. If Y/PY/NI to 2.4: Were these deviations from intended intervention balanced between groups?** |  |  |
| **2.6 Was an appropriate analysis used to estimate the effect of assignment to intervention?** |  | Y |
| **2.7 If N/PN/NI to 2.6: Was there potential for a substantial impact (on the result) of the failure to analyse participants in the group to which they were randomized?** |  |  |
| **Riskofbias judgement** |  | Low Risk |
| Optional: What is the predicted direction of bias due to deviations from intended interventions? |  | Unpredictable |

Domain 2: Risk of bias due to deviations from the intended interventions (*effect of adhering to intervention*)

| **Signalling questions** | **Comments** | **Response options** |
| --- | --- | --- |
| **2.1. Were participants aware of their assigned intervention during the trial?** |  | N |
| **2.2. Were carers and people delivering the interventions aware of participants' assigned intervention during the trial?** |  | Y |
| **2.3. [If applicable:] If Y/PY/NI to 2.1 or 2.2: Were important nonprotocol interventions balanced across intervention groups?** |  | PY |
| **2.4. [If applicable:] Were there failures in implementing the intervention that could have affected the outcome?** |  | NI |
| **2.5. [If applicable:] Was there nonadherence to the assigned intervention regimen that could have affected participants’ outcomes?** |  | PY |
| **2.6. If N/PN/NI to 2.3, or Y/PY/NI to 2.4 or 2.5: Was an appropriate analysis used to estimate the effect of adhering to the intervention?** |  | Y |
| **Riskofbias judgement** |  | Some concerns |
| Optional: What is the predicted direction of bias due to deviations from intended interventions? |  | Unpredictable |

Domain 3: Missing outcome data

| **Signalling questions** | **Comments** | **Response options** |
| --- | --- | --- |
| **3.1 Were data for this outcome available for all, or nearly all, participants randomized?** |  | Y |
| **3.2 If N/PN/NI to 3.1: Is there evidence that the result was not biased by missing outcome data?** |  | NA |
| **3.3 If N/PN to 3.2: Could missingness in the outcome depend on its true value?** |  | NA |
| **3.4 If Y/PY/NI to 3.3: Is it likely that missingness in the outcome depended on its true value?** |  | NA |
| **Riskofbias judgement** |  | Low |
| Optional: What is the predicted direction of bias due to missing outcome data? |  | Unpredictable |

Domain 4: Risk of bias in measurement of the outcome

| **Signalling questions** | **Comments** | **Response options** |
| --- | --- | --- |
| **4.1 Was the method of measuring the outcome inappropriate?** |  | N |
| **4.2 Could measurement or ascertainment of the outcome have differed between intervention groups?** |  | N |
| **4.3 If N/PN/NI to 4.1 and 4.2: Were outcome assessors aware of the intervention received by study participants?** |  | N |
| **4.4 If Y/PY/NI to 4.3: Could assessment of the outcome have been influenced by knowledge of intervention received?** |  |  |
| **4.5 If Y/PY/NI to 4.4: Is it likely that assessment of the outcome was influenced by knowledge of intervention received?** |  |  |
| **Riskofbias judgement** |  | Low |
| Optional: What is the predicted direction of bias in measurement of the outcome? |  | Unpredictable |

Domain 5: Risk of bias in selection of the reported result

| **Signalling questions** | **Comments** | **Response options** |
| --- | --- | --- |
| **5.1 Were the data that produced this result analysed in accordance with a prespecified analysis plan that was finalized before unblinded outcome data were available for analysis?** |  | Y |
| **Is the numerical result being assessed likely to have been selected, on the basis of the results, from...** |  |  |
| **5.2. ... multiple eligible outcome measurements (e.g. scales, definitions, time points) within the outcome domain?** |  | N |
| **5.3 ... multiple eligible analyses of the data?** |  | N |
| **Riskofbias judgement** |  | Low |
| Optional: What is the predicted direction of bias due to selection of the reported result? |  | Unpredictable |

Overall risk of bias

| **Riskofbias judgement** |  | Low |
| --- | --- | --- |
| Optional: What is the overall predicted direction of bias for this outcome? |  | Unpredictable |

**Supplemental Table 6.** Definitions of outcomes in included studies.

| Study/Year | 30-Day Mortality | 30-Day Stroke | 30-Day Major Bleeding | 30-Day Myocardial Infarction | Number of Days Prolonged Ventilation | 30-Day Acute Kidney Injury |
| --- | --- | --- | --- | --- | --- | --- |
| Afshar, 2023 | NR | Stroke, at one year | NR | NR | NR | NR |
| Agnino, 2022 | Operative mortality | Stroke/TIA | Transfusion of pRBC | NR | Prolonged mechanical ventilation | Acute renal failure |
| Agnino, 2019 | Death | NR | NR | NR | NR | NR |
| Ascaso, 2023 | In-hospital mortality | Permanent stroke | Patients transfused | NR | NR | AKI |
| Atluri, 2016 | Mortality at 30 days | Stroke | Re-exploration for bleeding | NR | Ventilatory support for > 24 hours | Renal failure |
| Balachandran, 2020 | NR | NR | NR | NR | NR | NR |
| Bellitti, 2014 | Intraoperative or in-hospital deaths | NR | NR | NR | NR | NR |
| Berdajs, 2023 | In-hospital mortality; death before discharge | NR | Reoperation for bleeding | Postoperative myocardial infarction | Intubation > 72 hours | Postoperative renal failure |
| Chemtob, 2022 | Hospital death | Stroke | Postoperative RBC transfusion | NR | NR | New-onset renal dialysis/new-onset renal failure |
| Chi, 2014 | Operative death | Stroke | NR | NR | NR | NR |
| D’Onofrio, 2022 | 30-day mortality | NR | Re-exploration for bleeding | NR | NR | Continuous venovenous hemofiltration |
| El-Andari, 2021 | All-cause mortality at 30 days | NR | NR | NR | NR | AKI |
| Franke, 2022 | Hospital mortality | Stroke | Re-exploration for bleeding | NR | NR | NR |
| Garatti, 2018 | 30-days mortality for isolated mitral valve repair | NR | NR | NR | NR | NR |
| Garcia Fuster, 2014 | In-hospital death | NR | NR | NR | NR | NR |
| Gerber, 2019 | Death | NR | NR | NR | NR | NR |
| Goldstone, 2015 | Death | Stroke | Transfusion | Myocardial infarction | Prolonged mechanical ventilation | Renal failure |
| Götte, 2022 | NR | Stroke before discharge | NR | Myocardial infarction before discharge | NR | Hemofiltration before discharge |
| Grapsa, 2015 | NR | NR | NR | NR | NR | NR |
| Grinberg, 2019 | Death | Stroke | Surgical revision for bleeding | NR | NR | NR |
| Güllü, 2021 | Perioperative mortality | Permanent neurological deficit | Bleeding | NR | NR | Renal failure requiring dialysis |
| Hasham, 2022 | 30-day mortality | Permanent stroke | Reoperation for bleeding | NR | Prolonged ventilation | Renal failure |
| Hayashi, 2021 | Hospital mortality | Cerebral infarction without disability | Reoperation for bleeding or transfusion | NR | NR | NR |
| Hu, 2021 | NR | NR | NR | NR | NR | NR |
| Huang, 2013 | Operative death | NR | NR | NR | NR | NR |
| Javadikasgari, 2017 | Postoperative in-hospital death | Postoperative in-hospital stroke | Postoperative in-hospital reoperation for bleeding | NR | NR | Postoperative in-hospital renal failure |
| Kamiya, 2017 | 30-day mortality | NR | Postoperative bleeding | NR | NR | NR |
| Kitai, 2014 | NR | NR | NR | NR | NR | NR |
| Kurlansky, 2023 | Operative mortality | NR | NR | NR | NR | NR |
| Lange, 2017 | Mortality 30 days | NR | NR | NR | NR | NR |
| Lee, 2018 | In-hospital mortality | NR | NR | NR | NR | NR |
| Levy, 2022 | Death during the postoperative period | Transient cerebral event | Blood transfusions required | NR | NR | NR |
| Li, 2022 | Death during the postoperative period | NR | Reoperation for bleeding | NR | NR | NR |
| Lio, 2017 | NR | NR | NR | NR | NR | NR |
| Ma, 2019 | Operative mortality | NR | Reoperation for bleeding | NR | Ventilation support > 5 days | Acute kidney failure |
| Magruder, 2016 | 30-day mortality | Stroke | NR | NR | Mechanical ventilation > 24 hours | Renal failure |
| Makarious, 2023 | Death within 30 days of operation or during the same hospitalization | NR | NR | NR | NR | NR |
| Mihaljevic, 2013 | Hospital mortality | Permanent stroke | NR | Perioperative myocardial infarction | Prolonged ventilation > 24 hours | Renal failure requiring dialysis |
| Moscarelli, 2018 | In-hospital death | Stroke/TIA | Reopening for bleeding | Postoperative myocardial infarction | NR | NR |
| Moscarelli, 2021 | 30-day mortality | Stroke/TIA | Reopening for bleeding | NR | Respiratory failure | Renal failure |
| Muneretto, 2015 | Hospital morality | Stroke | Transfusions of > 2 RBCs | Myocardial infarction | NR | NR |
| Murzi, 2014 | In-hospital mortality | Stroke | NR | Perioperative myocardial infarction | NR | Dialysis |
| Noack, 2019 | 30-day mortality | In hospital stroke | Reoperation for bleeding | In hospital myocardial infarction | NR | Dialysis for AKI |
| Panos, 2015 | 30-day mortality | Non-resolving cerebral ischemic episode | Reexploring for bleeding | NR | Prolonged ventilation | NR |
| Paranskaya, 2013 | In-hospital or 30-day mortality | Stroke | Intrathoracic hematoma or surgical revision | NR | Ventilation > 24 hours | Acute kidney failure |
| Perin, 2019 | In hospital mortality | In hospital stroke | Reoperation for bleeding | NR | Intubated for > 48 hours | NR |
| Petolat, 2023 | NR | NR | NR | NR | NR | NR |
| Ramzy, 2014 | NR | NR | Reoperation for bleeding | In hospital myocardial infarction | NR | Creatinine > 2.0 |
| Salihi, 2019 | 30-day mortality | 30-day stroke | Reoperation for bleeding | NR | NR | NR |
| Shibata, 2015 | 30-day mortality | NR | NR | NR | NR | NR |
| Smith, 2016 | Operative mortality | Permanent stroke | NR | NR | NR | Renal Failure |
| Solari, 2019 | Hospital Mortality | Ischemic stroke | Bleeding events requiring hospitalization | NR | NR | NR |
| Tomšič, 2019 | NR | NR | NR | NR | NR | NR |
| Uchimuro, 2014 | NR | NR | NR | NR | NR | NR |
| Vairo, 2023 | NR | NR | NR | NR | NR | NR |
| Van Leeuwen, 2013 | 30-day mortality | NR | Re-exploration for bleeding or tamponade | NR | NR | NR |
| Van Praet, 2022 | NR | NR | NR | NR | NR | NR |
| Wang, 2016 | 30-day mortality | NR | NR | NR | NR | NR |
| Westhofen, 2016 | In hospital mortality | In hospital stroke | Reoperation for bleeding | NR | NR | NR |
| Zorinas, 2019 | Postoperative mortality | Postoperative stroke | Postoperative re-exploration for bleeding | Postoperative myocardial infarction | NR | Renal failure (creatinine elevation by 150%) |

*AKI: Acute Kidney Injury; pRBC: Packed Red Blood Cells; RBC: Red Blood Cells; TIA: Transient Ischemic Attack*

| Study/Year | Long-term Kaplan Meier Mortality | Duration of follow-up | Long-term Kaplan Meier reoperation | Duration of follow-up | Long-term Kaplan Meier MR Recurrence | Duration of follow-up |
| --- | --- | --- | --- | --- | --- | --- |
| Afshar, 2023 | NR | NR | NR | NR | NR | NR |
| Agnino, 2022 | NR | NR | NR | NR | NR | NR |
| Agnino, 2019 | NR | NR | NR | NR | NR | NR |
| Ascaso, 2023 | NR | NR | NR | NR | Freedom from Severe MR | 6 years |
| Atluri, 2016 | NR | NR | NR | NR | NR | NR |
| Balachandran, 2020 | NR | NR | NR | NR | NR | NR |
| Bellitti, 2014 | Kaplan-Meier Survival | 12 years | Kaplan-Meier survival curve | 12 years | Freedom from recurrence of 2+ or greater mitral valve regurgitation | 12 years |
| Berdajs, 2023 | Freedom from All-cause mortality | 10 years | Freedom from MV re-operation | 10 years | NR | NR |
| Chemtob, 2022 | NR | NR | NR | NR | NR | NR |
| Chi, 2014 | NR | NR | NR | NR | NR | NR |
| D’Onofrio, 2022 | Survival | 5 years | NR | NR | Freedom from moderate-severe residual MR | 5 years |
| El-Andari, 2021 | NR | NR | NR | NR | NR | NR |
| Franke, 2022 | NR | NR | NR | NR | NR | NR |
| Garatti, 2018 | NR | NR | NR | NR | NR | NR |
| Garcia Fuster, 2014 | NR | NR | NR | NR | NR | NR |
| Gerber, 2019 | NR | NR | NR | NR | NR | NR |
| Goldstone, 2015 | Actuarial survival | 12.5 years | NR | NR | Actuarial development of moderate mitral regurgitation | 12.5 years |
| Götte, 2022 | NR | NR | Freedom from re-operation | 10 years | NR | NR |
| Grapsa, 2015 | NR | NR | NR | NR | NR | NR |
| Grinberg, 2019 | NR | NR | NR | NR | NR | NR |
| Güllü, 2021 | NR | NR | NR | NR | NR | NR |
| Hasham, 2022 | NR | NR | Freedom from mitral valve re-operation | 5 years | NR | NR |
| Hayashi, 2021 | NR | NR | NR | NR | NR | NR |
| Hu, 2021 | Survival | 12 years | NR | NR | Freedom from moderate or severe mitral regurgitation | 12 years |
| Huang, 2013 | NR | NR | NR | NR | NR | NR |
| Javadikasgari, 2017 | NR | NR | NR | NR | NR | NR |
| Kamiya, 2017 | NR | NR | NR | NR | NR | NR |
| Kitai, 2014 | NR | NR | NR | NR | NR | NR |
| Kurlansky, 2023 | Actuarial survival | 30 years | NR | NR | NR | NR |
| Lange, 2017 | Survival | 10 years | Reoperation | 10 years | NR | NR |
| Lee, 2018 | NR | NR | NR | NR | NR | NR |
| Levy, 2022 | NR | NR | NR | NR | NR | NR |
| Li, 2022 | NR | NR | NR | NR | NR | NR |
| Lio, 2017 | NR | NR | NR | NR | NR | NR |
| Ma, 2019 | Cumulative survival | 10.4 years | NR | NR | NR | NR |
| Magruder, 2016 | Survival probability | 10 years | NR | NR | NR | NR |
| Makarious, 2023 | Actuarial survival | 8 years | NR | NR | NR | NR |
| Mihaljevic, 2013 | NR | NR | NR | NR | NR | NR |
| Moscarelli, 2018 | NR | NR | NR | NR | Recurrent mitral regurgitation ≥2+ | 6 years |
| Moscarelli, 2021 | Survival Probability | 6 years | NR | NR | NR | NR |
| Muneretto, 2015 | NR | NR | Freedom from re-operation | 3 years | NR | NR |
| Murzi, 2014 | Cumulative survival | 10.4 years | Cumulative freedom for re-intervention | 5 years | NR | NR |
| Noack, 2019 | Survival | 10 years | Freedom from MV-related reoperation | 10 years | NR | NR |
| Panos, 2015 | Probability of survival | 7 years | Probability of freedom from mitral valve reoperation | 7 years | NR | NR |
| Paranskaya, 2013 | NR | NR | NR | NR | NR | NR |
| Perin, 2019 | NR | NR | NR | NR | NR | NR |
| Petolat, 2023 | NR | NR | NR | NR | NR | NR |
| Ramzy, 2014 | NR | NR | NR | NR | NR | NR |
| Salihi, 2019 | NR | NR | Freedom from re-operation | 4.2 years | Freedom from residual severe mitral regurgitation (MR) for all patients | 4.2 yearss |
| Shibata, 2015 | NR | NR | NR | NR | Overall freedom from moderate+ mitral regurgitation | 6 years |
| Smith, 2016 | NR | NR | NR | NR | NR | NR |
| Solari, 2019 | Survival | 15 years | Reoperation | 15 years | NR | NR |
| Tomšič, 2019 | Overall survival | 8 years | Freedom from re-intervention | 8 years | Freedom from recurrent MR | 8 years |
| Uchimuro, 2014 | NR | NR | NR | NR | Freedom from recurrent MR | 6 years |
| Vairo, 2023 | NR | NR | NR | NR | NR | NR |
| Van Leeuwen, 2013 | NR | NR | NR | NR | NR | NR |
| Van Praet, 2022 | NR | NR | NR | NR | NR | NR |
| Wang, 2016 | NR | NR | NR | NR | NR | NR |
| Westhofen, 2016 | NR | NR | NR | NR | NR | NR |
| Zorinas, 2019 | NR | NR | NR | NR | NR | NR |

**Supplemental Table 7.** References of Included Studies.

1. Afshar ZM, Sabzi F, Shirvani M, Salehi N, Nemati N, Kheradmand W, et al. Sex-Based Differences in One-Year Outcomes After Mitral Valve Repair for Infective Endocarditis. Braz J Cardiovasc Surg [Internet]. 2023 [cited 2024 Jan 9];38(5). Available from: https://cdn.publisher.gn1.link/bjcvs.org/pdf/0102-7638-rbccv-38-05-e20210333.pdf

2. Agnino A, Graniero A, Villari N, Roscitano C, Gerometta P, Albano G, et al. Evaluation of robotic-assisted mitral surgery in a contemporary experience. Journal of Cardiovascular Medicine. 2022 Jun;23(6):399–405.

3. Agnino A, Lanzone AM, Albertini A, Anselmi A. Follow-Up of the Novel Free Margin Running Suture Technique for Mitral Valve Repair. Thorac cardiovasc Surg. 2019 Oct;67(07):557–60.

4. Ascaso M, Sandoval E, Muro A, Barriuso C, Quintana E, Alcocer J, et al. Repair of mitral prolapse: comparison of thoracoscopic minimally invasive and conventional approaches. European Journal of Cardio-Thoracic Surgery. 2023 Aug 1;64(2):ezad235.

5. Atluri P, Stetson RL, Hung G, Gaffey AC, Szeto WY, Acker MA, et al. Minimally invasive mitral valve surgery is associated with equivalent cost and shorter hospital stay when compared with traditional sternotomy. The Journal of Thoracic and Cardiovascular Surgery. 2016 Feb;151(2):385–8.

6. Balachandran P, Schaff HV, Lahr BD, Nguyen A, Daly RC, Maltais S, et al. Preoperative left atrial volume index is associated with postoperative outcomes in mitral valve repair for chronic mitral regurgitation. The Journal of Thoracic and Cardiovascular Surgery. 2020 Sep;160(3):661-672.e5.

7. Bellitti R, Petrone G, Buonocore M, Nappi G, Santé P. Anatomic Reconstruction in Degenerative Mitral Valve Bileaflet Prolapse: Long-Term Results. The Annals of Thoracic Surgery. 2014 Feb;97(2):563–8.

8. Berdajs D, Miazza J, Koechlin L, Gahl B, Reuthebuch O, Eckstein F. Minimally Invasive Nonresectional Mitral Valve Repair Long-term Results. Canadian Journal of Cardiology. 2023 Jul;39(7):990–6.

9. Chemtob RA, Wierup P, Mick SL, Javorski MJ, Burns DJP, Blackstone EH, et al. A conservative screening algorithm to determine candidacy for robotic mitral valve surgery. The Journal of Thoracic and Cardiovascular Surgery. 2022 Oct;164(4):1080–7.

10. Chi NH, Huang CH, Huang SC, Yu HY, Chen YS, Wu IH. Robotic mitral valve repair in infective endocarditis. Journal of Thoracic Disease. 2014;6(1).

11. D’Onofrio A, Mastro F, Nadali M, Fiocco A, Pittarello D, Aruta P, et al. Transapical beating heart mitral valve repair versus conventional surgery: a propensity-matched study. Interactive CardioVascular and Thoracic Surgery. 2022 Jun 15;35(1):ivac053.

12. EL-Andari R, Bozso SJ, Kang JJH, Boe D, Hong Y, Gill RS, et al. The effects of body mass index on long-term outcomes and cardiac remodeling following mitral valve repair surgery. Int J Obes. 2021 Dec;45(12):2679–87.

13. Franke UFW, Huether F, Ghinescu M, Gaviria MO, Rufa MI, Albert M, et al. Robotically assisted mitral valve surgery—experience during the restart of a robotic program in Germany. Ann Cardiothorac Surg. 2022 Nov;11(6):596–604.

14. Garatti A, Canziani A, Parolari A, Castelvecchio S, Guazzi M, Daprati A, et al. Long-term results of suture annuloplasty for degenerative mitral valve disease: a propensity-matched analysis. Journal of Cardiovascular Medicine. 2018 Jan;19(1):22–8.

15. García Fuster R, Martín E, Paredes F, Mena A, Cánovas S, Gil O, et al. Artificial chordae in the setting of complex mitral valve repair: early outcomes using the folding leaflet technique. Interact CardioVasc Thorac Surg. 2014 May;18(5):586–95.

16. Gerber W, Sanetra K, Drzewiecka-Gerber A, Jankowska-Sanetra J, Bochenek A, Zembala M, et al. Echocardiographic evaluation of the results of minimally invasive repair of asymptomatic mitral regurgitation. Kardiol Pol. 2019 Jan 22;77(1):56–8.

17. Goldstone AB, Cohen JE, Howard JL, Edwards BB, Acker AL, Hiesinger W, et al. A “Repair-All” Strategy for Degenerative Mitral Valve Disease Safely Minimizes Unnecessary Replacement. The Annals of Thoracic Surgery. 2015 Jun;99(6):1983–91.

18. Götte J, Zittermann A, Hakim-Meibodi K, Hata M, Schramm R, Bleiziffer S, et al. Long-Term Clinical Outcome in Elderly Patients Undergoing Mitral Valve Repair. Thorac Cardiovasc Surg. 2022 Mar;70(02):093–9.

19. Grapsa J, Zimbarra Cabrita I, Jakaj G, Ntalarizou E, Serapheim A, Demir OM, et al. Strain balance of papillary muscles as a prerequisite for successful mitral valve repair in patients with mitral valve prolapse due to fibroelastic deficiency. European Heart Journal - Cardiovascular Imaging. 2015 Jan 1;16(1):53–61.

20. Grinberg D, Cottinet PJ, Thivolet S, Audigier D, Capsal JF, Le MQ, et al. Measuring chordae tension during transapical neochordae implantation: Toward understanding objective consequences of mitral valve repair. The Journal of Thoracic and Cardiovascular Surgery. 2019 Sep;158(3):746–55.

21. Güllü AÜ, Senay S, Kocyigit M, Zencirci E, Akyol A, Degirmencioglu A, et al. An analysis of the learning curve for robotic‐assisted mitral valve repair. J Card Surg. 2021 Feb;36(2):624–8.

22. Hashim SW, Mcmahon SR, Vaitkeviciute IK, Collazo S, Hashim IM, Loya DS, et al. Propensity-matched comparison of right mini-thoracotomy versus median sternotomy for isolated mitral valve repair. J Cardiovasc Surg [Internet]. 2022 Dec [cited 2024 Jan 9];63(6). Available from: https://www.minervamedica.it/index2.php?show=R37Y2022N06A0724

23. Hayashi Y, Nakamura Y, Hirano T, Ito Y, Watanabe T. Cumulative sum analysis for the learning curve of minimally invasive mitral valve repair. Heart Vessels. 2021 Oct;36(10):1584–90.

24. Hu X, Jiang W, Li H, Yan G, Wang Y. Timing of Valve Repair for Asymptomatic Mitral Regurgitation and Preserved Left Ventricular Function. The Annals of Thoracic Surgery. 2021 Mar;111(3):862–70.

25. Huang H lei, Xie X jing, Fei H wen, Xiao X jun, Liu J, Zhuang J, et al. Real-time three-dimensional transesophageal echocardiography to predict artificial chordae length for mitral valve repair. J Cardiothorac Surg. 2013 Dec;8(1):137.

26. Javadikasgari H, Suri RM, Tappuni B, Lowry AM, Mihaljevic T, Mick S, et al. Robotic mitral valve repair for degenerative posterior leaflet prolapse. Ann Cardiothorac Surg. 2017 Jan;6(1):27–32.

27. Kamiya H, Akhyari P, Minol JP, Ites AC, Weinreich T, Sixt S, et al. Simple technique of repair for Barlow syndrome with posterior resection and chordal transfer via minimally invasive approach: primary experience in a consecutive series of 22 patients. Gen Thorac Cardiovasc Surg. 2017 Jul;65(7):374–80.

28. Kitai T, Okada Y, Shomura Y, Tani T, Kaji S, Kita T, et al. Timing of valve repair for severe degenerative mitral regurgitation and long-term left ventricular function. The Journal of Thoracic and Cardiovascular Surgery. 2014 Nov;148(5):1978–82.

29. Kurlansky PA, Traad EA, Ebra G. Mitral Repair vs Replacement for Degenerative Mitral Regurgitation in Patients Aged ≥65 Years. The Annals of Thoracic Surgery. 2023 Oct;116(4):736–42.

30. Lange R, Voss B, Kehl V, Mazzitelli D, Tassani-Prell P, Günther T. Right Minithoracotomy Versus Full Sternotomy for Mitral Valve Repair: A Propensity Matched Comparison. The Annals of Thoracic Surgery. 2017 Feb;103(2):573–9.

31. Lee HA, Lin CY, Chen YC, Chen SW, Nan YY, Liu KS, et al. Surgical interventions of isolated active mitral valve endocarditis: Predisposing factors and impact of neurological insults on final outcome. Medicine. 2018 Mar;97(11):e0054.

32. Levy F, Wautot F, Dommerc C, Iacuzio L, Civaia F, Marcacci C, et al. Echocardiographic characteristics of non‐resectional ring‐only valve repair in mitral valve prolapse. Echocardiography. 2022 Apr;39(4):612–9.

33. Li G, Li P, Liu S, You B. Follow-Up of Robotic Mitral Valve Repair: A Single Tertiary Institution Experience in China. Hussein AF, editor. Computational and Mathematical Methods in Medicine. 2022 May 21;2022:1–5.

34. Lio A, Miceli A, Ferrarini M, Glauber M. Mitral valve repair using a prosthetic ring with chordal sizing system: a modified technique in the presence of myxomatous leaflets. European Journal of Cardio-Thoracic Surgery. 2017 Oct 1;52(4):820–2.

35. Ma W, Shi W, Wu W, Ye W, Kong Y, Zhu D, et al. Elevated gradient after mitral valve repair: The effect of surgical technique and relevance of postoperative atrial fibrillation. The Journal of Thoracic and Cardiovascular Surgery. 2019 Mar;157(3):921-927.e3.

36. Magruder JT, Collica S, Belmustakov S, Crawford TC, Grimm JC, Cameron DE, et al. Predictors of Late-Onset Atrial Fibrillation Following Isolated Mitral Valve Repairs in Patients With Preserved Ejection Fraction: AF After MVR. J Card Surg. 2016 Aug;31(8):486–92.

37. Makarious Laham M, Easo J, Szczechowicz M, Roosta-Azad M, Weymann A, Ruhparwar A, et al. Five-year follow-up of mitral valve repair versus replacement: a propensity score analysis. J Cardiothorac Surg. 2023 Jan 16;18(1):27.

38. Mihaljevic T, Pattakos G, Gillinov AM, Bajwa G, Planinc M, Williams SJ, et al. Robotic Posterior Mitral Leaflet Repair: Neochordal Versus Resectional Techniques. The Annals of Thoracic Surgery. 2013 Mar;95(3):787–94.

39. Moscarelli M, Di Bari N, Fattouch K, Brigiani MS, Bonifazi R, Nasso G, et al. Minimally Invasive Mitral Valve Repair for Standalone Secondary Mitral Regurgitation. Heart, Lung and Circulation. 2021 Mar;30(3):431–7.

40. Moscarelli M, Terrasini N, Nunziata A, Punjabi P, Angelini G, Solinas M, et al. A Trial of Two Anesthetic Regimes for Minimally Invasive Mitral Valve Repair. Journal of Cardiothoracic and Vascular Anesthesia. 2018 Dec;32(6):2562–9.

41. Muneretto C, Bisleri G, Bagozzi L, Repossini A, Berlinghieri N, Chiari E. Results of minimally invasive, video-assisted mitral valve repair in advanced Barlow’s disease with bileaflet prolapse†. European Journal of Cardio-Thoracic Surgery. 2015 Jan;47(1):46–51.

42. Murzi M, Miceli A, Cerillo AG, Di Stefano G, Kallushi E, Farneti P, et al. Training Surgeons in Minimally Invasive Mitral Valve Repair: A Single Institution Experience. The Annals of Thoracic Surgery. 2014 Sep;98(3):884–9.

43. Noack T, Marin Cuartas M, Kiefer P, Garbade J, Pfannmueller B, Seeburger J, et al. Isolated Mitral Valve Repair in Patients with Reduced Left Ventricular Ejection Fraction. ATCS. 2019;25(6):326–35.

44. Panos A, Vlad S, Milas F, Myers PO. Is minimally invasive mitral valve repair with artificial chords reproducible and applicable in routine surgery? Interact CardioVasc Thorac Surg. 2015 Jun;20(6):707–11.

45. Paranskaya L, D’Ancona G, Bozdag-Turan I, Akin I, Kische S, Turan GR, et al. Percutaneous vs Surgical Repair of Mitral Valve Regurgitation: Single Institution Early and Midterm Outcomes. Canadian Journal of Cardiology. 2013 Apr;29(4):452–9.

46. Perin G, Shaw M, Pingle V, Palmer K, Al-Rawi O, Ridgway T, et al. Use of an automated knot fastener shortens operative times in minimally invasive mitral valve repair. annals. 2019 Sep;101(7):522–8.

47. Petolat E, Theron A, Resseguier N, Fabre C, Norscini G, Badaoui R, et al. Prognostic value of forward flow indices in primary mitral regurgitation due to mitral valve prolapse. Front Cardiovasc Med. 2023 Feb 23;10:1076708.

48. Ramzy D, Trento A, Cheng W, De Robertis MA, Mirocha J, Ruzza A, et al. Three hundred robotic-assisted mitral valve repairs: The Cedars-Sinai experience. The Journal of Thoracic and Cardiovascular Surgery. 2014 Jan;147(1):228–35.

49. Salihi S. Is transapical off-pump neochord implantation a safe and effective procedure for mitral valve repair? Anatol J Cardiol [Internet]. 2019 [cited 2024 Jan 9]; Available from: https://anatoljcardiol.com/jvi.aspx?un=AJC-17055

50. Shibata T, Kato Y, Motoki M, Takahashi Y, Morisaki A, Nishimura S, et al. Mitral valve repair with loop technique via median sternotomy in 180 patients. European Journal of Cardio-Thoracic Surgery. 2015 Mar;47(3):491–6.

51. Smith AAH, Newman RV, Thompson PA, Farkas SI, Wynne J. Mitral Valve Repair Outcomes in a Community Hospital: A Retrospective Analysis. Heart, Lung and Circulation. 2016 May;25(5):499–504.

52. Solari S, De Kerchove L, Tamer S, Aphram G, Baert J, Borsellino S, et al. Active infective mitral valve endocarditis: is a repair-oriented surgery safe and durable?†. European Journal of Cardio-Thoracic Surgery. 2019 Feb 1;55(2):256–62.

53. Tomšič A, Hiemstra YL, Van Brakel TJ, Versteegh MIM, Marsan NA, Klautz RJM, et al. Outcomes of Valve Repair for Degenerative Disease in Patients With Mitral Annular Calcification. The Annals of Thoracic Surgery. 2019 Apr;107(4):1195–201.

54. Uchimuro T, Tabata M, Saito K, Shibayama K, Watanabe H, Fukui T, et al. Post-repair coaptation length and durability of mitral valve repair for posterior mitral valve prolapse. Gen Thorac Cardiovasc Surg. 2014 Apr;62(4):221–7.

55. Vairo A, Gaiero L, Marro M, Russo C, Bolognesi M, Soro P, et al. New Echocardiographic Parameters Predicting Successful Trans-Ventricular Beating-Heart Mitral Valve Repair with Neochordae at 3 Years: Monocentric Retrospective Study. JCM. 2023 Feb 22;12(5):1748.

56. Van Leeuwen WJ, Head SJ, De Groot-de Laat LE, Geleijnse ML, Bogers AJJC, Van Herwerden LA, et al. Single-centre experience with mitral valve repair in asymptomatic patients with severe mitral valve regurgitation†. Interactive CardioVascular and Thoracic Surgery. 2013 Jun;16(6):731–7.

57. Van Praet KM, Kofler M, Hirsch S, Akansel S, Hommel M, Sündermann SH, et al. Factors associated with an unsuccessful fast-track course following minimally invasive surgical mitral valve repair. European Journal of Cardio-Thoracic Surgery. 2022 Sep 2;62(4):ezac451.

58. Wang Y, Gao CQ, Shen YS, Wang G. Echocardiographic Follow-up of Robotic Mitral Valve Repair for Mitral Regurgitation due to Degenerative Disease. Chinese Medical Journal. 2016 Sep 20;129(18):2199–203.

59. Westhofen S, Conradi L, Deuse T, Detter C, Vettorazzi E, Treede H, et al. A matched pairs analysis of non-rib-spreading, fully endoscopic, mini-incision technique versus conventional mini-thoracotomy for mitral valve repair. Eur J Cardiothorac Surg. 2016 Dec;50(6):1181–7.

60. Zorinas A, Lipnevicius A, Bleizgytė V, Janušauskas V, Liekienė D, Budra M, et al. Comparison of early postoperative results between conventional and transapical mitral valve repair. pwki. 2019;15(4):439–45.
